# Local structure propensities in disordered proteins from cross-correlated NMR spin relaxation

**DOI:** 10.1007/s10858-025-00460-3

**Published:** 2025-02-26

**Authors:** Daniel Braun, Clemens Kauffmann, Andreas Beier, Irene Ceccolini, Olga O. Lebedenko, Nikolai R. Skrynnikov, Robert Konrat

**Affiliations:** 1 Department of Computational and Structural Biology, University of Vienna, Campus Vienna Biocenter 5, Vienna, 1030 Vienna, Austria; 2 Laboratory of Biomolecular NMR, St. Petersburg State University, St. Petersburg, Russia; 3 Department of Chemistry, Purdue University, West Lafayette, IN USA

**Keywords:** Disordered proteins, Structural propensity, Nuclear magnetic resonance, Cross-correlated relaxation, Molecular dynamics

## Abstract

Structurally diverse ensembles of intrinsically disordered proteins or regions are difficult to determine, because experimental observables usually report a conformational average. Therefore, in order to infer the underlying distribution, a set of experiments that measure different aspects of the system is necessary. In principle, there exists a set of cross-correlated relaxation (CCR) rates that report on protein backbone geometry in a complementary way. However, CCR rates are hard to interpret, because geometric information is encoded in an ambiguous way and they present themselves as a convolute of both structure and dynamics. Despite these challenges, CCR rates analyzed within a suitable statistical framework are able to identify conformations in structured proteins. In the context of disordered proteins, we find that this approach has to be adjusted to account for local dynamics via including an additional CCR rate. The results of this study show that CCR rates can be used to characterize structure propensities also in disordered proteins. Instead of using an experimental reference structure, we employed computational spectroscopy to calculate CCR rates from molecular dynamics (MD) simulations and subsequently compared the results to conformations as observed directly in the MD trajectory.

**Supplementary Information:**

The online version contains supplementary material available at 10.1007/s10858-025-00460-3.

## Introduction

Intrinsically disordered proteins (IDPs) and intrinsically disordered regions (IDRs) do not have a single rigid structure, but rather exist as an ensemble of dynamically interconverting conformations. This poses a formidable challenge for conventional structural biology and requires novel theoretical and experimental approaches. Despite their long-known prevalence and importance in a multitude of physiological and, in particular, pathogenic processes, understanding of their often context-dependent behavior is still in its infancy (Wright and Dyson 1999; Dunker et al. 2001; Babu et al. 2011; Holehouse and Kragelund 2024). Therefore, the development of experimental approaches that are able to detect local and global structure propensities of disordered proteins is crucial for the establishment of a "sequence-ensemble-function" (Holehouse and Kragelund 2024) relationship and would constitute a milestone in structural biology.

Nuclear magnetic resonance (NMR) spectroscopy offers a number of experiments that are well suited for the investigation of highly dynamic biological systems under near-native conditions. For example, cross-correlated spin relaxation (CCR) rates are among the more convoluted but at the same time potentially most informative ones. They are interference effects that occur in the presence of multiple relaxation mechanisms of equal tensor rank. Importantly, these interactions can be observed even if different spins are affected. This "spooky action at a distance" (Vögeli and Vugmeyster 2019) can be exploited to investigate the structural degrees of freedom between the observed nuclei. Applications to proteins were first proposed by Reif et al., who probed the backbone dihedral angle $\psi$ by measuring CCR between sequential $^{15}\hbox {N-}^1\hbox {H}^N$ and $^{13}\hbox {C}^\alpha \hbox {-}^1\hbox {H}^\alpha$ dipole pairs (Reif et al. 1997). Similar experiments probing different spins and dihedral angles were developed soon after (Yang et al. 1997; Pelupessy et al. 1999; Kloiber and Konrat 2000; Skrynnikov et al. 2000; Kloiber and Konrat 2000; Chiarparin et al. 2000; Pelupessy et al. 2003) and have been further established in the following decades (Vögeli and Vugmeyster 2019). However, thus far, applications have remained limited to folded proteins. The common perception of the scientific community has been that CCR rates encode too much information for the application to IDPs. As phrased by Ferrage and coworkers: "The broad conformational space of IDPs makes the wide variety of cross-correlated relaxation methods developed for folded proteins difficult to interpret in disordered proteins, as the effects of average relative orientations (structure) and correlation times (dynamics) are difficult to deconvolute" (Bolik-Coulon et al. 2019, p. 72).

This work aims to resolve this long-lasting challenge. In a previous study (Kauffmann et al. 2021), we could demonstrate that a joint analysis of different CCR rates allowed us to characterize the backbone dihedral angle distribution of a folded protein such as ubiqutin, extending even to the disordered C-terminal tail. For that system, the main source of variation was found to stem from the ensemble averages of angular dependencies of CCR rates instead of their dynamics (Kauffmann et al. 2021). Here, we propose that a similar approach can be applied to IDPs, with the addition of including a CCR with known amplitude that is used as a measure for local dynamics in the analysis.

To test this, we employ a self-consistent approach based on molecular dynamics (MD). Using MD trajectories of a structured and a disordered protein with a combined simulation time of 62.5 microseconds, we calculated a set of ten CCR rates (eight remote rates, two rates with known amplitude) per residue. These rates were then used to reconstruct the underlying backbone dihedral angle distributions, also known as Ramachandran plots (Ramachandran and Sasisekharan 1968). Comparing these to the "true" Ramachandran plots (as observed directly in MD), we can show that CCR rates can be used to characterize conformational properties and structure propensities in disordered proteins.

## Theory

Cross-correlated relaxation (CCR) describes the interference between two simultaneously active relaxation mechanisms with equal tensor rank. For the most commonly considered mechanisms in solution-state NMR, dipolar (DP) and chemical shift anisotropy (CSA), these interference effects are encoded by time correlation functions (TCFs) of the form

1 $$\begin{aligned} C_{{\varvec{a}},{\varvec{b}}}(t) = \langle P_2({\varvec{a}}(0) \cdot {\varvec{b}}(t)) \rangle \end{aligned}$$

in isotropic solution. $P_2(x) = 1.5x^2-0.5$ is the second order Legendre polynomial, ${\varvec{a}}$ and ${\varvec{b}}$ are motional vectors corresponding to dipolar unit vectors or principal components of CSA tensors, and angled brackets denote the ensemble average. For simplicity, we omit the contributions of time-dependent fluctuations of dipolar distances and/or CSA tensor geometries for now. These effects are addressed in more detail in the “Methods” section.

Identifying the dot product as the cosine of the projection angle $\theta _{{\varvec{a}},{\varvec{b}}}$, we see that the TCF encodes the average degree of correlation in $P_2(\cos \theta _{{\varvec{a}},{\varvec{b}}}$) between ${\varvec{a}}$ and ${\varvec{b}}$ as a function of *t*. For the considered “remote” CCR interferences, which do not share any spin between the two relaxation mechanisms, the observable rate $\Gamma$ is proportional to the spectral density at zero frequency, or, in case of a fully anisotropic CSA tensor, a linear combination of zero spectral densities. Considering the simpler case as an example for now, we obtain:

2 $$\begin{aligned} \Gamma _{{\varvec{a}},{\varvec{b}}} \propto J_{{\varvec{a}},{\varvec{b}}}(0) = \int _0^\infty C_{{\varvec{a}},{\varvec{b}}}(t) \,dt \end{aligned}$$

Thus, the CCR rate encodes both structural and dynamical information about the relation between ${\varvec{a}}$ and ${\varvec{b}}$, albeit in rather convoluted fashion. Formally, one can separate the two components to obtain a more intuitive expression:

3 $$\begin{aligned} J_{{\varvec{a}},{\varvec{b}}}(0) = C_{{\varvec{a}},{\varvec{b}}}(0) \int _0^\infty \frac{C_{{\varvec{a}},{\varvec{b}}}(t)}{C_{{\varvec{a}},{\varvec{b}}}(0)} \,dt = \langle P_2({\varvec{a}}(0) \cdot {\varvec{b}}(0)) \rangle \tau _{{\varvec{a}},{\varvec{b}}} \end{aligned}$$

Dividing the TCF by its amplitude $C_{{\varvec{a}},{\varvec{b}}}(0)$, it is normalized, i.e. it starts off at 1. The integral of the normalized TCF is defined as the correlation time $\tau _{{\varvec{a}},{\varvec{b}}}$; it represents all relaxation mechanisms that affect $C_{{\varvec{a}},{\varvec{b}}}$ and is often called the "effective correlation time" in the context of NMR. The amplitude is the initial (“static”) population average at $t = 0$. By adopting a model backbone geometry with $\phi$ and $\psi$ as the only conformational degrees of freedom, we can approximate $C_{{\varvec{a}},{\varvec{b}}}(0)$ as an average over an underlying dihedral angle distribution ${\varvec{p}}_{\phi ,\psi }$:

4 $$\begin{aligned} & \langle P_2({\varvec{a}}(0) \cdot {\varvec{b}}(0)) \rangle = \langle P_2(\cos \theta _{{\varvec{a}},{\varvec{b}}}(\phi ,\psi )) \rangle \nonumber \\ & \approx \sum _{\phi ,\psi } p_{\phi ,\psi } P_2(\cos \theta _{{\varvec{a}},{\varvec{b}}}(\phi ,\psi )) \end{aligned}$$

Anticipating the subsequent numerical implementation, we already denote the distribution ${\varvec{p}}_{\phi ,\psi }$ as a finite probability vector; its entries $p_{\phi ,\psi }$ act as the weights in a weighted sum defining the ensemble average. In the following, subscripts ${\varvec{a}}$, ${\varvec{b}}$ are dropped for convenience or replaced when indicating specific correlated components.

To make sense of CCR rates we must know either *C*(0) or $\tau$. For folded proteins, both components can be reasonably approximated: ${\varvec{p}}_{\phi ,\psi }$ could be known from a structural model; $\tau$ mostly follows the rotational tumbling time of the protein. The case of a flexible backbone is more complex: *C*(0) results from an unknown and broadly populated ${\varvec{p}}_{\phi ,\psi }$, while $\tau$ does not follow a simple analytical model, encoding site-specific local motions, i.e. dynamics is heterogeneous along the protein backbone. In fact, the angular degrees of freedom governing the amplitude of the TCF might also affect its decay (Kämpf et al. 2018). Thus in disordered proteins, separation of *C*(0) and $\tau$ is more involved than in structured proteins.

### Retrieving the structural component

The investigated CCR rates $\Gamma$ are defined by (linear combinations of) *J*(0), i.e. time integrals from 0 to $\infty$ of TCFs with specific correlated components ${\varvec{a}},{\varvec{b}}$, combined with the corresponding pre-factors (see Appendix). In principle, CCR rates are convoluted observables of a structural component $A_{\Gamma }$, representing the rates when *C*(0) is used instead of time integrals *J*(0), and a dynamical component $\tau _{\Gamma }$, described by the decay of the underlying TCFs, i.e. the time integrals of the normalized TCFs, see Eq. (3). Unfortunately, in an experimental context neither $A_{\Gamma }$ nor $\tau _{\Gamma }$ are directly accessible, but only their product $\Gamma$. Therefore, in systems with heterogeneous dynamics, it is necessary to define an approximation for $\tau _{\Gamma }$ to be able to predict ${\varvec{p}}_{\phi ,\psi }$ from a set of $A_{\Gamma }$ of different remote CCR rates ${\Gamma }$.

For a folded protein such as Ubiquitin, ${\varvec{p}}_{\phi ,\psi }$ can be extracted with reasonable accuracy for a broad range of assumed $\tau _{\Gamma }$; all CCR rates were assumed to experience the same, slightly down-scaled tumbling time of the protein. While rates predicted from this simple model deviated considerably from experiment, ${\varvec{p}}_{\phi ,\psi }$ could still be extracted with sufficient accuracy. Essentially, it was found that the main source of variation between rates lies in their amplitudes, not in their decay (Kauffmann et al. 2021).

In IDPs, correlation times are expected to be more heterogeneous, i.e. spin relaxation is more local in nature and does not follow the overall tumbling of the protein (Kämpf et al. 2018). Therefore, we need to identify how to best measure these local dynamics. For auto-correlated rates and other rates with known amplitude, the correlation time can be extracted directly from experiment. A straightforward and commonly measured option is the relaxation of the NH dipolar vector; its zero spectral density can be mapped through various experimental strategies (Kadeřávek et al. 2014) and provides a direct, residue-resolved measure of the local correlation time. Alternatively, we recently proposed a set of experiments to probe the correlation time related to the the $C'C_{\alpha }$ bond (Loth et al. 2005; Kauffmann et al. 2021). Presuming for now that such estimates are indeed capable of approximating the correlation times of nearby remote CCR interactions, we can construct a suitable model to extract ${\varvec{p}}_{\phi ,\psi }$.

### Accounting for ensemble averaging

First, we will formalize the general problem in the language of Bayesian statistics. Given a set of CCR rates $\Gamma _1,\ldots ,\Gamma _j$, denoted as a vector $\varvec{\Gamma }$, we want to infer the underlying distribution ${\varvec{p}}_{\phi ,\psi }$. Assuming for now that the correlation times are known, the observed CCR rates are a function of ${\varvec{p}}_{\phi ,\psi }$ alone. We then write this problem as a conditional probability, i.e. we assign any realization of ${\varvec{p}}_{\phi ,\psi }$ a probability *P* given the data $\varvec{\Gamma }$. Rearranging this expression,

5 $$\begin{aligned} P({\varvec{p}}_{\phi ,\psi }\vert \varvec{\Gamma })&= \dfrac{P({\varvec{p}}_{\phi ,\psi }\cap \varvec{\Gamma })}{P(\varvec{\Gamma })} = \dfrac{\dfrac{P({\varvec{p}}_{\phi ,\psi }\cap \varvec{\Gamma })}{P({\varvec{p}}_{\phi ,\psi })}P({\varvec{p}}_{\phi ,\psi })}{P(\varvec{\Gamma })} \nonumber \\ &= \dfrac{P(\varvec{\Gamma }\vert {\varvec{p}}_{\phi ,\psi })P({\varvec{p}}_{\phi ,\psi })}{P(\varvec{\Gamma })} \end{aligned}$$

yields Bayes’ theorem. It provides a way to calculate the quantity of interest, the posterior probability $P({\varvec{p}}_{\phi ,\psi }\vert \varvec{\Gamma })$, by evaluating the likelihood $P(\varvec{\Gamma }\vert {\varvec{p}}_{\phi ,\psi })$ and the prior $P({\varvec{p}}_{\phi ,\psi })$. Since we are only interested in comparing the relative probabilities of different ${\varvec{p}}_{\phi ,\psi }$, i.e. we take the observed data $\varvec{\Gamma }$ as given, we can omit the evidence $P(\varvec{\Gamma })$. Bayes’ theorem then reads: *posterior* $\propto$ *likelihood* $\times$ *prior*. Evidently, Bayesian inference does not single out one ’solution’, rather it produces a probability distribution over different realizations of ${\varvec{p}}_{\phi ,\psi }$; a result that is very general but also quite cumbersome. Often, one is interested in obtaining simpler and more convenient point estimates for $P({\varvec{p}}_{\phi ,\psi }\vert \varvec{\Gamma })$.

For the likelihood, we will adhere to the commonly made assumption of uncorrelated Gaussian errors:

6 $$\begin{aligned} P(\varvec{\Gamma }\vert {\varvec{p}}_{\phi ,\psi }) \propto \exp \bigg (-\sum _{j=1}^m \frac{( \Gamma _j ({{{\varvec{p}}_{\phi ,\psi }}}) -\Gamma _j)^2}{2\sigma _j^2}\bigg ) \end{aligned}$$

The smaller (the sum of) the $\chi ^2$ between the functions $\Gamma _j ({{{\varvec{p}}_{\phi ,\psi }}})$ and the observed rates $\Gamma _j$ are, the higher is the probability of the underlying ${\varvec{p}}_{\phi ,\psi }$. The variances $\sigma _j^2$ quantify the experimental uncertainty (Kauffmann et al. 2021) associated with each rate (and thus are set to 1 for the purposes of this work).

The prior $P({\varvec{p}}_{\phi ,\psi })$ requires more consideration. Encoding our intuition and knowledge about the problem, it contains the solutions we allow for a priori. For example, in our first implementation(Kloiber et al. 2002), we presumed a rigid fold, i.e. only point-like distributions ${\varvec{p}}_{\phi ,\psi }$ were considered, effectively negating all ensemble-averaging effects. Preferring no one ($\phi$, $\psi$)-pair over another, i.e. assuming a uniform prior, the posterior could then be visualized and evaluated simply as a function of $\phi$ and $\psi$.

To model a more flexible backbone, the prior $P({\varvec{p}}_{\phi ,\psi })$ must allow for more broadly populated distributions. Not only do we need to account for ensemble averaging effects explicitly, we also must anticipate the increased ambiguity they might introduce: A more general prior might contain many different ${\varvec{p}}_{\phi ,\psi }$ with similar $\chi ^2$; the likelihood flattens, so to speak. With the intention to derive a simple point estimate for ${\varvec{p}}_{\phi ,\psi }$, the prior must be shaped accordingly.

A common strategy is to employ an entropy prior, which can be derived from first principles (Skilling 1989; Gull 1989) or introduced more ad hoc (Hummer and Köfinger 2015):

7 $$\begin{aligned} P({\varvec{p}}_{\phi ,\psi }) \propto \exp \bigg (-T\sum _{\phi ,\psi } p_{\phi ,\psi } \log \frac{p_{\phi ,\psi }}{q_{\phi ,\psi }}\bigg ) \end{aligned}$$

The closer ${\varvec{p}}_{\phi ,\psi }$ is to ${\varvec{q}}_{\phi ,\psi }$ in terms of its relative entropy or Kullback–Leibler divergence $D_{KL}$, the higher is its probability. Similarly to the variances $\sigma _j^2$, the scaling factor or “temperature” *T* quantifies the confidence in ${\varvec{q}}_{\phi ,\psi }$, i.e. the prior expectation for ${\varvec{p}}_{\phi ,\psi }$ before observing any data.

The practical reason for employing this prior lies in the convexity of $D_{KL}$. A distribution over possible distributions is quite cumbersome to evaluate. To simplify further, we pick the maximum of $P({\varvec{p}}_{\phi ,\psi }\vert \varvec{\Gamma })$, i.e. the most likely realization, as a representative point approximation in a Maximum A Posteriori (MAP) estimate. Noting that $\max (\exp ) = \max (\log (\exp )) = \min (-\log (\exp ))$, we obtain a familiar optimization problem:

8 $$\begin{aligned} \min _{{\varvec{p}}_{\phi ,\psi }}\; D_{KL}({\varvec{p}}_{\phi ,\psi }\Vert {\varvec{q}}_{\phi ,\psi }) \cdot T + \sum _{j=1}^m \frac{( \Gamma _j ({{{\varvec{p}}_{\phi ,\psi }}}) -\Gamma _j)^2}{2\sigma _j^2} \end{aligned}$$

We obtain a simple regularized $\chi ^2$-fit using an entropy penalty. Not by coincidence, this expression corresponds to the well-known Maximum Entropy (MaxEnt) formalism first proposed by Gull and Daniell (1978). The above generalization in Bayesian terms was first derived by Skilling (1989) and Gull (1989) and recently rediscovered by Hummer and Köfinger in the context of structural biology(Hummer and Köfinger 2015; Köfinger et al. 2019). Practical differences generally revolve around the treatment of the free parameter *T*. Here, *T* will simply be optimized to achieve a sensible balance between $\chi ^2$ and $D_{KL}$ using an L-curve heuristic (Miller 1970; Hansen 1992). Again, instead of a fully Bayesian treatment (Bryan 1990), we resort to simpler point estimates.

### Self-consistent validation

The central question of this study is whether the proposed approach can yield a good approximation for ${\varvec{p}}_{\phi ,\psi }$. Since this is expected to partially depend on the characteristics of the investigated system, we consider both folded proteins and IDPs.

For folded proteins like Ubiquitin, experimentally derived ensemble representations are available (Lange et al. 2008); assessing the validity of the proposed approach is thus straightforward(Kauffmann et al. 2021). For IDPs, one lacks experimental “ground truth” and must resort to an asserted representation of the ensemble and its dynamics. Arguably, molecular dynamics (MD) simulations provide the best structural and dynamical models for IDPs available to date (Rauscher et al. 2015; Robustelli et al. 2018; Piana et al. 2020). For the intended purpose of validation, MD simulations can serve as a self-contained framework that extends beyond the limits of analytical models.

In a first step, CCR rates are calculated from simulated trajectories and, in analogy to experimental procedure, subsequently processed to yield ${\varvec{p}}_{\phi ,\psi }$. Next, these results are compared to a reference distribution ${\varvec{r}}_{\phi ,\psi }$ as observed directly in the underlying trajectories, assessing the validity and limitations of the proposed protocol (Kämpf et al. 2018; Bremi and Brüschweiler 1997; Case 2002; Prompers and Brüschweiler 2002).

Force fields for biomolecular and particularly IDP simulations have drastically improved in recent years. Here, we employ an advanced model called des-amber (Robustelli et al. 2018) that was developed to cover a broad range of systems. More specifically, it is suitable for both ordered and disordered proteins and was further refined with an emphasis on non-bonded potentials. While the motivation for the development of the last iteration of this force field family was a better description of intermolecular interactions between biological macromolecules, we believe that it can have additional merits compared to previous force fields even in the case of a single-chain disordered system, considering the frequent occurrence of transient interactions between remote residues.

It is worth noting that quantitative agreements with experiments are by no means a strict requirement for the intended purpose of this study. As long as the characteristics of the simulation can be considered a realistic representation of the generic structural dynamics of proteins/IDPs, the general workflow of predicting ${\varvec{p}}_{\phi ,\psi }$ from CCR rates can be validated in a fully self-consistent manner, namely by comparison to the reference distribution ${\varvec{r}}_{\phi ,\psi }$ calculated from the same underlying simulation data.

## Methods

### Conceptual approach

As lined out in the Theory section, protein backbone $\phi$,$\psi$-distributions from a molecular dynamics (MD) ensemble ${\varvec{r}}_{\phi ,\psi }$ are compared with distributions predicted from CCR rates ${\varvec{p}}_{\phi ,\psi }$, which are themselves calculated from the same MD ensemble. This self-consistent approach eliminates possible sources of uncertainty and errors that might have been present in previous studies comparing data from different experiments for the case of a folded protein (Kloiber et al. 2002; Kauffmann et al. 2021) and offers the means to quantify the influence of different components (e.g., the employed dynamical model) on the quality of the final prediction. More importantly, however, it allows us to extend the analysis to the case of IDPs, where no adequate experimental "ground truth" for their structural propensity states is available at this point.

### MD simulations

#### MD setup

Simulations of 76-residue-long globular protein Ubiquitin (UBQ) and 25-residue-long disordered N-terminal tail of histone protein H4 (NH4) were carried out with the simulation package GROMACS ver. 2022 (Berendsen et al. 1995; Abraham et al. 2015; Páll 2015) and a state-of-the-art force field for structured and disordered proteins called des-amber (Piana et al. 2020).

The inital structure of Ubiquitin 1UBQ (Vijay-Kumar et al. 1987) from the RCSB protein data bank (Berman et al. 2000) was stripped of all water molecules. Missing hydrogens were patched and it was solvated in a cubic box containing 13,370 water molecules together with 38 potassium ($\hbox {K}^+$) and 38 chloride ($\hbox {Cl}^-$) ions (roughly corresponding to physiological osmolarity). For NH4, a starting structure was generated via PEP-FOLD (Thevenet et al. 2012; Shen et al. 2014), which was then protonated with H++ (Gordon et al. 2005) to yield the same total charge (+8) as in Kämpf et al. (2018). Subsequently, the protein was solvated in a cubic box containing 12,988 water molecules, 37 potassium ions and 45 chloride ions (to achieve a net charge of zero for the whole system, again at physiological osmolarity).

Throughout the simulation protocol, the Verlet cutoff-scheme (Páll and Hess 2013) for non-bonded interactions was employed with a force-switch cut-off between 1.0 and 1.2 nm together with dispersion corrections for pressure and energy. All simulations were performed under periodic boundary conditions. For the treatment of long-range electrostatic interactions, the smooth particle mesh Ewald method (Darden et al. 1993; Essmann et al. 1995) was used with cubic grid spacing of 0.16 nm as an upper bound and interpolation order 4. Integration of atomic coordinates was handled by the leapfrog scheme (Amini et al. 1987) at a time step of 2 fs. All bonds involving hydrogens were constrained by the LINCS algorithm (Hess et al. 1997). The average temperature was held at 300 K with a Nose-Hoover thermostat (Nosé 1984; Hoover 1985) across the whole system with a coupling constant of 1 ps. In *NPT* simulations, the Parrinello-Rahman barostat (Parrinello and Rahman 1981; Nosé and Klein 1983) maintained an average pressure of 1 bar with a time constant of 2 ps.

After a steepest descent minimization of 50,000 steps, equilibration runs of 100 ps in the *NVT* ensemble were carried out, during which protein coordinates were held constant. Then, protein coordinates were released and the systems were equilibrated in the *NPT* ensemble for 10 ns and 1$\upmu$s for UBQ and NH4, respectively. Production runs in the *NPT* ensemble had a length of 12.5 $\upmu$s for the UBQ system and 50 $\upmu$s for the NH4 system. During production, coordinates of all protein atoms were recorded at every picosecond.

#### MD analysis

Ramachandran plots were constructed by first extracting time series of $\phi _i$ and $\psi _i$ for each residue *i* from the UBQ and NH4 trajectories with a 100 ps graining using the MDAnalysis framework (Michaud-Agrawal et al. 2011; Gowers et al. 2016). These time series were then converted to the two-dimensional probability distribution by entering pairs of $\phi _i$ and $\psi _i$ into bins of size $10^{\circ }\times$ $10^{\circ }$.

CCR rates $\Gamma$ (with an index denoting different kinds of experiments) are calculated as

9 $$\begin{aligned} \Gamma _{ab,cd}= & \frac{2}{5}\bigg (\frac{\mu _0\hbar }{4\pi }\bigg )^2 \gamma _a \gamma _b \gamma _c \gamma _d \int _0^\infty \bigg \langle \frac{P_2(\textbf{ab}(0)\cdot \textbf{cd}(t))}{r_{ab}(0)^3 r_{cd}(t)^3}\bigg \rangle \,dt \end{aligned}$$

10 $$\begin{aligned} \Gamma _{ab,u}= & \frac{4}{15}\frac{\mu _0\hbar }{4\pi } \gamma _a \gamma _b \gamma _u B_0 \sum _{k=x,y} (\sigma ^u_{kk}-\sigma ^u_{zz})\int _0^\infty \bigg \langle \frac{P_2(\textbf{ab}(0)\cdot \mathbf {kk_u}(t))}{r_{ab}(0)^3}\bigg \rangle \,dt \end{aligned}$$

11 $$\begin{aligned} \Gamma _{u,v}= & \frac{8}{45} \gamma _u \gamma _v B_0^2 \sum _{\begin{array}{c} k=x,y \\ l=x,y \end{array}} (\sigma ^u_{kk}-\sigma ^u_{zz})(\sigma ^v_{ll}-\sigma ^v_{zz})\int _0^\infty \big \langle P_2(\textbf{kk}_u(0)\cdot \textbf{ll}_v(t))\big \rangle \,dt \end{aligned}$$

where $\gamma$ is the gyromagnetic ratio; $B_0$ is the magnetic field strength (set to 800 MHz operational frequency in this study); $\mu _0$ is the vacuum permeability; $\hbar$ is the reduced Planck constant; *a*, *b*, *c* and *d* are nuclei subject to dipolar coupling; *r* is the distance between two nuclei, the dipolar unit vector connecting them is indicated by an arrow; *u* and *v* are nuclei with CSA; $\sigma _{xx}$, $\sigma _{yy}$, $\sigma _{zz}$ are the principal components of the CSA tensor (in ppm), the corresponding eigenvectors are indicated in bold. We assume an average backbone-$C'$ CSA tensor geometry with $(\sigma _{xx}, \sigma _{yy}, \sigma _{zz})$ = (249.4, 191.1, 87.9) ppm and $\alpha _{C'N,xx}$ = $37^{\circ }$, and an average backbone-*N* CSA tensor geometry with $\sigma _{xx}-\sigma _{zz} \approx \sigma _{xx} - \sigma _{yy}$ = 170 ppm and $\alpha _{N N^H,xx}$ = $20^{\circ }$ (Loth et al. 2005; Kauffmann et al. 2021). The specific expressions for each CCR rate are compiled in the Appendix.

In order to calculate time correlation functions (TCFs) of Eqs. (9)-(11) from simulation data, time series of the corresponding vectors were extracted from trajectories again using the MDAnalysis framework. CSA vectors were defined so that $\textbf{zz}$ lies perpendicular to the plane instantaneously spanned by $\mathbf {C'N}$ and $\mathbf {C'O}$ for the backbone-${C'}$-CSA and by $\mathbf {NH^N}$ and $\mathbf {NC'}$ for the backbone-*N*-CSA (Loth et al. 2005). According to Eqs. (17)–(26), the respective time series were correlated using the convolution theorem with fast Fourier transforms (Flannery et al. 1992). To increase sampling quality of the cross-correlation functions, the components were also correlated in reversed order and the resulting TCF was taken as the average of the two. Instantaneous inter-nuclear distances in the denominator of TCFs were only considered for the $\mathbf {H_{i}^{\alpha }H_{i+1}^{N}}$ (Eq. (21) and (10)), while for the rest (vectors defined by bonded atoms) the mean over the whole trajectory was used.

CCR rates $\Gamma$ are defined via *J*(0) and thus were computed via integration of these TCFs in combination with their respective prefactors, see Eqs. 9–11 and 17–26. The good sampling quality of the TCFs and narrow time interval (1 ps) allowed us to perform numerical integration instead of relying on (multi-)exponential fits, which is prone to introducing artifacts. In order to minimize statistical noise descending from finite simulation time, the numerical integral was taken over the initial part of TCFs, i.e. in the UBQ system from 0 to 20 ns. At this point, all calculated correlation functions are converged well towards zero (see Fig. S5). From the overall tumbling time of UBQ we can derive that less than 1% of the initial correlation must be present at this delay time. For the much smaller but disordered protein NH4 we choose an upper integration bound of 10 ns, which encompasses an additional margin of safety. As can be seen in Fig. S3, also in the NH4 system, all TCFs converge well towards zero within the chosen time interval.

### Tools for predicting $\phi$, $\psi$-distributions from CCR rates

#### Definition of dynamical proxy

In the simulation context of this study, we can readily derive the correlation time $\tau _{\Gamma }$ for each rate $\Gamma$ by simply setting the amplitude of the respective TCF to 1 prior to integration. Note that TCFs depending on the $C'$-CSA are defined as a linear combination of component-TCFs weighted by $(\sigma _{xx}, \sigma _{yy}, \sigma _{zz})$. Furthermore, we can directly extract the purely structural component as $A_{\Gamma }=\Gamma /\tau _{\Gamma }$, which is representative of *C*(0) times its respective prefactors.

While $A_{\Gamma }$ can be used to assess the employed statistical framework to predict $\phi ,\psi$-distributions in isolation (i.e. not subject to biases from dynamics), in an experimental setting, $A_{\Gamma }$ is not directly accessible, but only the convolute of structure and dynamics in the form of rates $\Gamma$. Hence, it is crucial to be able to approximate $A_{\Gamma }$ of remote CCR rates, for which we need an experimentally accessible quantity that approximates $\tau _{\Gamma }$. As lined out in the Theory section, we propose to derive a measure for local dynamics from $\Gamma _{N,NH^N}$ (Eq. 25) describing the correlation between backbone-*N*-CSA and backbone-$\mathbf {NH^N}$ or, alternatively, from $\Gamma _{C',C'C_{\alpha }}$ (Eq. (26)) describing correlation between backbone-$C'$-CSA and backbone-$\mathbf {C'C^{\alpha }}$ (Kauffmann et al. 2021). Since the average relative orientation between their correlated components is known, i.e. their amplitude is known, these can be treated like auto-correlated rates where correlation time $\tau$ is experimentally accessible and can be used as an estimate for localized dynamics. In the context of predicting $\phi$, $\psi$-distributions, we propose to use a linear combination of two rates that belong to the two encompassing peptide planes of the respective residue *i* as

12 $$\begin{aligned} \tau ^i_{N,NH^N} = \frac{\Gamma _{N_{i},N_{i}H^{N}_{i}} + \Gamma _{N_{i+1},N_{i+1}H^{N}_{i+1}}}{2~A_{N,NH^N}} \end{aligned}$$

or

13 $$\begin{aligned} \tau ^i_{C^{'},C^{'}C^{\alpha }} = \frac{\Gamma _{C'_{i-1},C'_{i-1}C^{\alpha }_{i-1}} + \Gamma _{C'_{i},C'_{i}C^{\alpha }_{i}}}{2~A_{C^{'},C^{'}C^{\alpha }}}. \end{aligned}$$

where *A* are the known values of *C*(0) combined with their respective prefactors:

14 $$\begin{aligned} A_{N,NH^N}= & \frac{4}{15} \gamma _N B_0 \frac{\mu _0\hbar }{4\pi } \gamma _N \gamma _H (\sigma ^N_{xx}-\sigma ^N_{zz}) P_2 (NH^N \cdot xx^N) \end{aligned}$$

15 $$\begin{aligned} A_{C^{'},C^{'}C^{\alpha }}= & \frac{4}{15} \gamma _C B_0 \frac{\mu _0\hbar }{4\pi } \gamma _C^{2} \sum _{k=x,y} (\sigma ^{C'}_{kk}-\sigma ^{C'}_{zz}) P_2(C'C^{\alpha } \cdot kk^{C'}) \end{aligned}$$

Each remote CCR rate $\Gamma$ can then be divided by $\tau _{N,NH^N}$ or $\tau _{C^{'},C^{'}C^{\alpha }}$ to approximate their respective $A_{\Gamma }$. The statistical framework described in the following takes remote CCR rates $\Gamma$ together with a measure for dynamics, either one of the dynamical proxies $\tau _{N,NH^N}$ or $\tau _{C^{'},C^{'}C^{\alpha }}$ as an input for predicting ${\varvec{p}}_{(\phi ,\psi )}$.

#### Statistical framework for predicting $\phi$, $\psi$-distributions from CCR rates

**Fig. 1:**
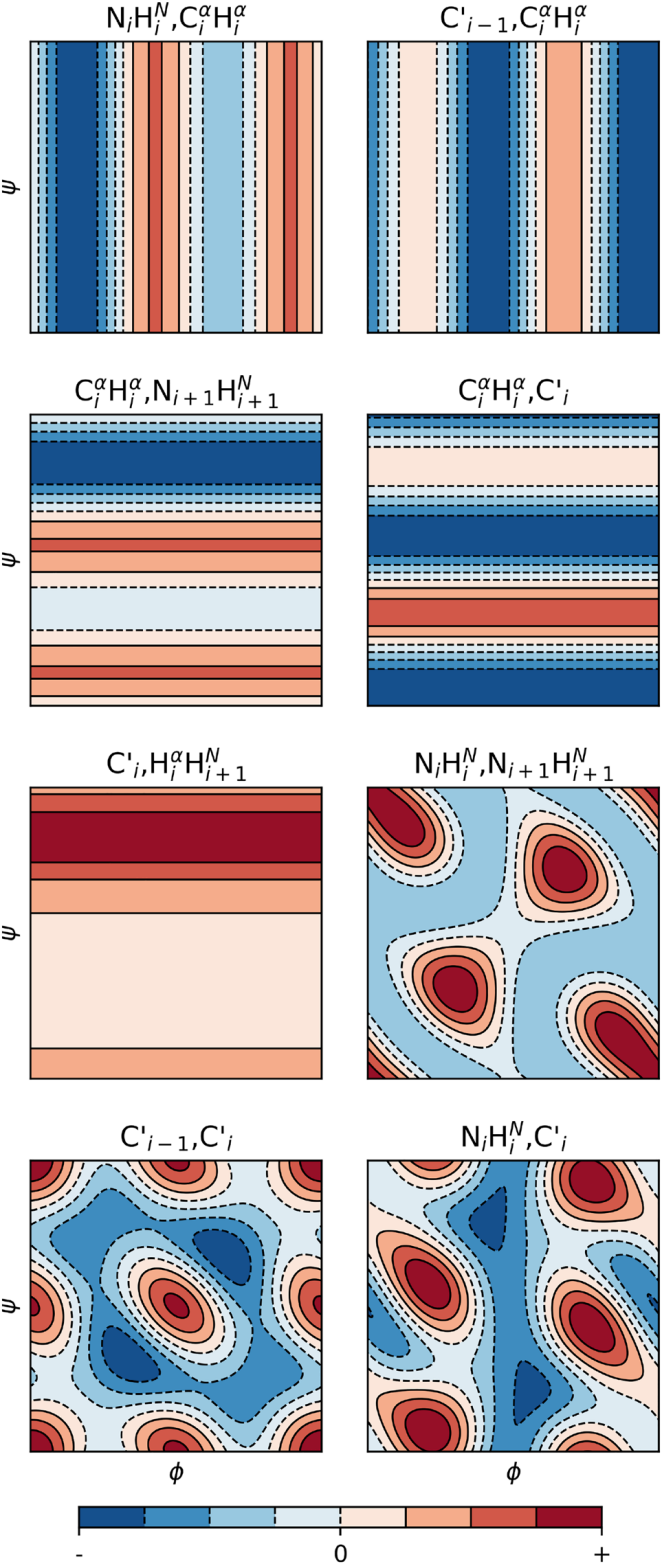
Dependencies of remote CCR rates’ structural component $A_{\Gamma }$ on backbone angles $\phi ,\psi$ in an otherwise rigid model. All axes range from − 180°to 180°

According to Eq. (6), we need to define a function $\Gamma _j({\varvec{p}}(\phi ,\psi ))$ for each rate *j* that will be compared to the observed rate $\Gamma _j$. Equation (3) splits $J(0) \propto \Gamma$ into a structural and dynamical component. For the latter the dynamical proxy defined in the previous Sect. (Definition of dynamical proxy) is used, while the structural component is estimated by rotating an otherwise rigid model backbone (see Table S1) over $\phi$ and $\psi$ in one-degree steps. The same CSA tensor geometry as in Sect. “MD analysis” is assumed; the $NH^N$ and $C^{\alpha }H^{\alpha }$ distances are adjusted post hoc to match the average values obtained from the MD simulation, 1.01 and 1.09 Å. The resulting angular dependencies for each rate are depicted in Fig. 1. The prior distribution

${\varvec{q}}_{\phi ,\psi }$ is the same as used in (Kauffmann et al. 2021): the random coil prior of Mantsyzov et al. (2014) compiled onto a 360 × 360 grid ($1^{\circ }$ resolution), discarding ill-defined terminal segments as well as glycine and proline residues. The regularization parameter *T* in Eq. (8) was sampled between 0.01 and 3 in varying step sizes. Upon visual inspection of predictions together with corresponding L-curves and for the sake of comparability, *T* was set to 1 for all predictions in this work.

### Data visualisation and metrics

Before visualisation, distributions predicted from computed CCR rates ${\varvec{p}}_{\phi ,\psi }$ were averaged to a 36x36 grid ($10^{\circ }$ resolution), matching the reference distributions ${\varvec{r}}_{\phi ,\psi }$ (extracted directly from simulation data).

As a quantitative measure of the similarity between ${\varvec{p}}_{\phi ,\psi }$ and ${\varvec{r}}_{\phi ,\psi }$ we chose the Jensen-Shannon (JS) divergence

16 $$\begin{aligned} D_{JS}({\varvec{p}}_{\phi ,\psi }\Vert {\varvec{r}}_{\phi ,\psi }) = \frac{1}{2} \sum _{\phi ,\psi } \left( {p}_{\phi ,\psi } \log _2{\frac{{p}_{\phi ,\psi }}{{m}_{\phi ,\psi }}} + {r}_{\phi ,\psi } \log _2{\frac{{r}_{\phi ,\psi }}{{m}_{\phi ,\psi }}}\right) \end{aligned}$$

where ${m}_{\phi ,\psi }$ is the element-wise average $0.5*({p}_{\phi ,\psi } + {r}_{\phi ,\psi })$. We use the form of this metric with the binary logarithm so that it scales between 0 (in case of identical distributions) and 1 (in case of completely non-overlapping probabilities).

## Results and discussion

In an experimental context, the extraction of ${\varvec{p}}(\phi ,\psi )$ from a set of measured CCR rates can be viewed as a two-step process. First, we need to establish a suitable method for the deconvolution of structure and dynamics inherent to remote CCR rates (see Eqs. (2)and (3)). Therefore, the first subchapter deals with the identification of an optimal proxy for the rate-specific correlation time $\tau _{\Gamma }$. In the second subchapter, remote CCR rates and the dynamical proxy identified in the first subchapter together are used to predict ${\varvec{p}}(\phi ,\psi )$.

For the purposes of this paper, CCR rates are calculated from MD simulations and used for predictions ${\varvec{p}}(\phi ,\psi )$ (backbone angle distributions), which are then compared to ${\varvec{r}}(\phi ,\psi )$, the reference distribution calculated directly from the very same simulations. In the following, we focus on representative residues of the disordered protein NH4. The full range of data for all residues from both NH4 and the globular protein UBQ can be found in the SI.

### Identification of optimal dynamical proxy

As a first candidate to approximate the rate-specific correlation time $\tau _{\Gamma }$ we investigate the exchange-free rate $\Gamma _{N,NH^N}$ (Loth et al. 2005; Kadeřávek et al. 2014), which behaves similar to the auto-relaxation of the NH vector (see “Methods”). The correlation time $\tau _{N,NH^N}$ can be derived via dividing $\Gamma _{N,NH^N}$ (Eq. 25) by $A_{N,NH^N}$ (Eq. 14), also in an experimental context. Note that we define $\tau _{N,NH^N}$ as the average correlation time of both peptide planes encompassing the respective residue, see Eq. (12), and $\Gamma _{N,NH^N}$ is experimentally accessible via a linear combination of longitudinal and transversal rates (Kadeřávek et al. 2014; Kauffmann et al. 2021).

**Fig. 2:**
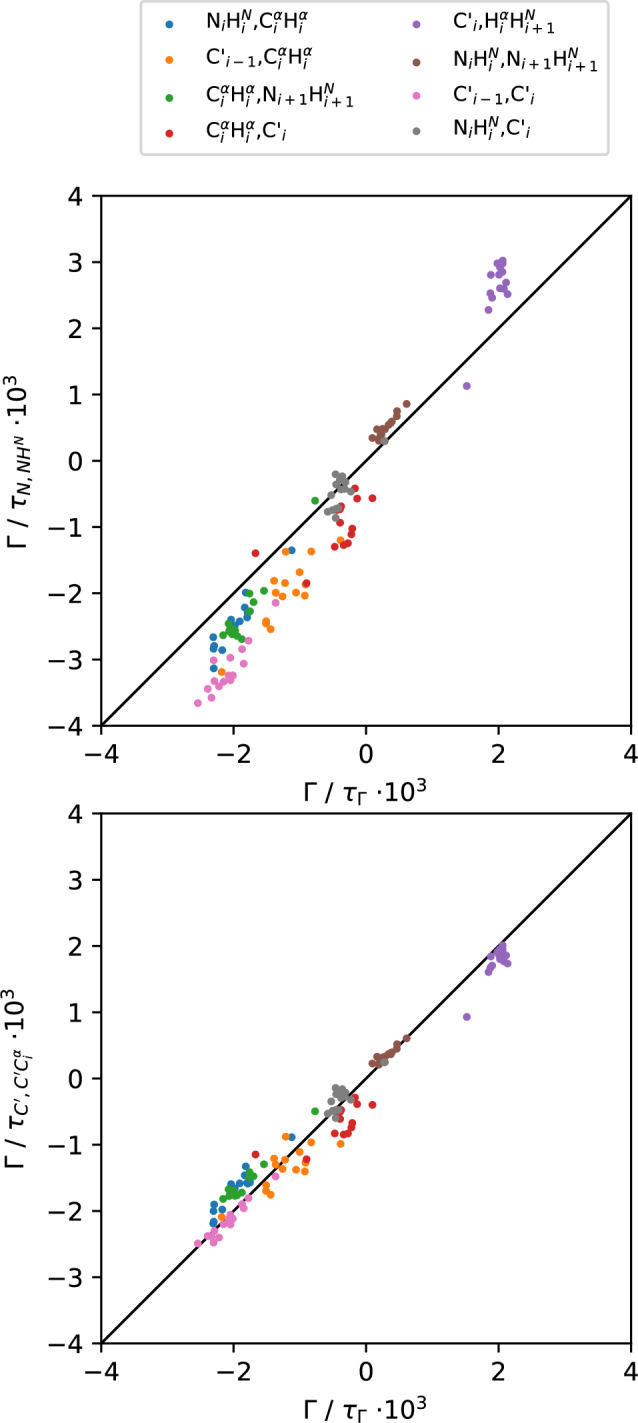
NH4: rates’ structural component $A_{\Gamma }=\Gamma /\tau _{\Gamma }$ vs. approximated structural component** a** $\Gamma /\tau _{N,NH^N}$, see Eq. (12) and** b** $\Gamma /\tau _{C',C'C^{\alpha }}$, see Eq. (13). Both distributions have an $R^2=0.97$. The slope of a linear fit through (0, 0) is 1.37 for (**a**) and 0.93 for (**b**)

Figure 2a shows sets of remote CCR rates for all eligible residues of protein NH4. Rates $\Gamma$ divided by their own correlation time $\tau _{\Gamma }$ yield the structural components $A_{\Gamma }$. They are plotted on the x-axis against the experimentally accessible observable $\Gamma / \tau _{N,NH^N}$ on the y-axis. While the correlation of this approximation is remarkably good ($R^2=0.97$), the slope of a linear fit through all points and (0,0) is 1.37. A similar behavior is observed in the structured protein UBQ, where $R^2=0.99$ and the slope is 1.13, see Fig. S2a. This suggests that $\tau _{N,NH^N}$ tends to be too small compared to the actual correlation time $\tau _{\Gamma }$ for the majority of rates. This behavior is observed in both structured and disordered systems, albeit more pronounced in the latter.

In a similar fashion, the rate $\Gamma _{C',C'C_{\alpha }}$ Eq. (26) can be used to extract exchange-free correlation times in an experimental context (Ceccolini et al. 2024; Kauffmann et al. 2021). Again, we can derive $A_{C',C'C_{\alpha }}$ due to the assumed fixed average mutual geometry of the $C'C_{\alpha }$ dipole vector and $C'$-CSA tensor components (Loth et al. 2005). Like before, we define $\tau _{C',C'C_{\alpha }}$ as the average correlation time of both peptide planes encompassing the respective residue, see Eq. (13).

Looking at Fig. 2b, the approximation via $\tau _{C',C'C_{\alpha }}$ yields the same $R^2=0.97$ as with $\tau _{N,NH^N}$. However, in absolute terms the approximated structural components are much closer to the true $A_{\Gamma }$, reflected by a slope of 0.93 in NH4. For the structured case of UBQ, the approximation via $\tau _{C',C'C_{\alpha }}$ results in a slope of exactly 1.00 (see Fig. S2b).

Precisely due to the remote nature of remote CCR rates, $\tau _{\Gamma }$ is expected to be governed by overall dynamics of a residue. Therefore, it seems logical that $\tau _{C',C'C_{\alpha }}$ proves to be a better approximation of $\tau _{\Gamma }$ than $\tau _{N,NH^N}$. This can be rationalized by $\Gamma _{C',C'C_{\alpha }}$ not being subjected to the librational motions specific to the backbone–NH bond (Salvi et al. 2017) and importantly, a diversification of relaxation processes. More precisely, the $C'$-CSA tensor is sensitive to motions in multiple spatial directions and thus $\Gamma _{C',C'C_{\alpha }}$ is more representative of potentially anisotropic local dynamics, which seems to be particularly pronounced in IDPs, supported by recent experimental evidence (Ceccolini et al. 2024). This, in turn, implies that some remote rates—the ones that are particularly sensitive to the degrees of freedom specific to the peptide plane, i.e. include correlations with the backbone-$H^N$ - should be slightly too small in absolute terms when normalized via $\tau _{C',C'C_{\alpha }}$, see Fig 2b. In fact, defining a $\tau _{opt}=0.7 \cdot \tau _{C',C'C_{\alpha }} + 0.3 \cdot \tau _{N,NH^N}$ for normalizing all remote rates that include the backbone-$H^N$ in NH4 leads to $R^2=0.99$ and a slope of 0.99, see Fig. S1. While these weightings are most likely generalizable, they could be affected by the investigated protein system and might also slightly change with a different model. Considering other sources of uncertainty in the process of obtaining backbone angle distributions, $\tau _{C',C'C_{\alpha }}$ constitutes a sufficiently accurate approximation on its own for now. Ergo, with simplicity in mind, we continue our analysis with $\tau _{C',C'C_{\alpha }}$ as the dynamical proxy.

### Prediction of backbone angle distributions

Predicted backbone angle distributions ${\varvec{p}}(\phi ,\psi )$ are obtained from remote CCR rates and the dynamical proxy (here $\tau _{C',C'C_{\alpha }}$, see previous section) via a MaxEnt framework according to Eq. (8), see “Methods” section. All rates were calculated from MD simulations of the disordered protein NH4 and the structured protein UBQ via numeric integration of converged time series, see Sect. “MD simulations” and Figs. S3 and S5. For all predictions, a random coil prior (Mantsyzov et al. 2014) as well as a regularization parameter of 1 is used, see Sect. “Statistical framework for predicting phi,psi-distributions from CCR rates”. We compare predicted distributions ${\varvec{p}}(\phi ,\psi )$ against reference distributions ${\varvec{r}}(\phi ,\psi )$, which were calculated from the same MD simulations.

**Fig. 3:**
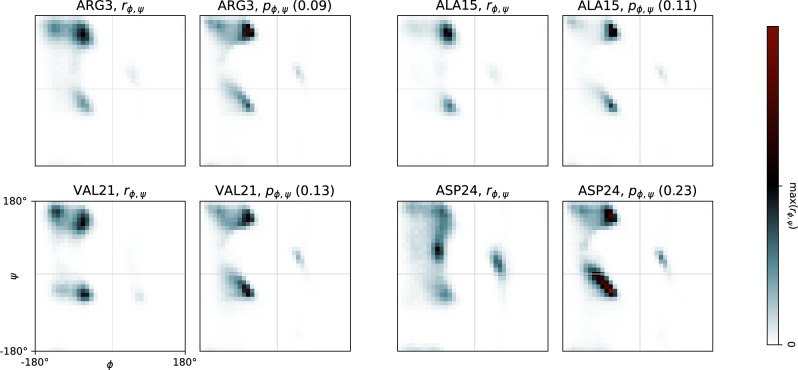
NH4 $\phi$,$\psi$-distributions for selected residues. For each of the four residues, the reference distribution ${\varvec{r}}_{\phi ,\psi }$ extracted directly from simulation data is shown on the left, and the distribution predicted from the set of CCR rates (with $\tau _{C',C'C^{\alpha }}$ as dynamical proxy) computed from the same simulation data is shown on the right. The color scale is defined by the range of the respective ${\varvec{r}}_{\phi ,\psi }$. Note that predicted probabilities exceeding this range are indicated with red shading. For all predictions, the Jensen-Shannon divergence (between 0 and 1) with respect to ${\varvec{r}}_{\phi ,\psi }$ is provided (see “Methods”)

A set of four representative residues from the disordered protein NH4 is shown in Fig. 3. As a first and central observation we note that the disordered nature per se is identified correctly for all residues in NH4 (also see Fig. S4), notably while successfully predicting structured states in UBQ with the very same setup (see Fig. S6). The average JS divergence (Eq. (16)) of all NH4 residues is 0.13.

In the following, we look at individual residues in greater detail. The reference distribution ${\varvec{r}}(\phi ,\psi )$ of ARG3 in NH4 populates all common regions: $\alpha$-helix ($\alpha$), $\beta$-sheet ($\beta$), polyproline II and left-handed helix. All of these are present in the prediction ${\varvec{p}}(\phi ,\psi )$ with remarkably accurate sub-probabilities for each region resulting in a JS div. of only 0.09. The next example ALA15 exhibits decreased probability density in $\beta$, which is also reflected in the respective ${\varvec{p}}(\phi ,\psi )$ with a JS div. of 0.11. Residue VAL21 exhibits higher probability density in $\alpha$ than the previous two examples, as well as a second weak $\alpha$-peak and an almost unpopulated left-handed helix region. While the higher probability density of $\alpha$ is clearly reflected in ${\varvec{p}}(\phi ,\psi )$, the exact shape of $\alpha$ and the disappearance of left-handed helix are not. Instead, the shape of the prior prevails, resulting in a slightly higher JS div. of 0.13. This problem becomes more severe in the last example ASP24, where ${\varvec{r}}(\phi ,\psi )$ is quite unusual, with a maximum in the otherwise poorly populated region between $\alpha$ and $\beta$. Here, the MaxEnt framework is not able to find weights that sufficiently alter the prior distribution, or the information content of remote rates is simply not high enough to converge to the correct distribution. On the other hand, a relatively higher population density in the left-handed helix region is represented well in ${\varvec{p}}(\phi ,\psi )$. Despite obvious flaws in the prediction, the JS divergence of 0.23 is relatively low, because the approximate nature of a disordered residue is reflected in both ${\varvec{r}}(\phi ,\psi )$ and the employed prior and the partially optimized ${\varvec{p}}(\phi ,\psi )$. Finally, using an alternative dynamical proxy $\tau _{N,NH^N}$ or the structural components directly, i.e. normalization via $\tau _{\Gamma }$ (see Fig. S4), we observe little variations in the prediction, implying that deviations from ${\varvec{r}}(\phi ,\psi )$ stem mostly from the employed statistical framework or a lack of information content in the set of remote rates.

Predictions with an identical setup for UBQ also yield meaningful distributions, with the maximum of the distributions reflected correctly for practically all residues (see Fig. S6). Broader distributions in the flexible tail of UBQ are also reflected well in predictions. In some cases, the framework is not able to eliminate all irrelevant regions from the random-coil prior, which was already observed in a previous study (Kauffmann et al. 2021). This leads to an on average higher JS divergence of 0.26. As a side note, the fact that some residues show the lowest JS div. with $\tau _{N,NH^N}$ as a dynamical proxy in UBQ can be explained by error compensation (originating from a random-coil prior combined with artificially sharper distributions due to $\tau _{N,NH^N}$).

## Conclusion

In this MD-simulation study, we have demonstrated that a set of eight remote CCR rates combined with a suitable proxy for local dynamics from one more CCR rate can be used to successfully predict backbone angle distributions (Ramachandran plots) of residues in both structured and disordered proteins. For the structured case, the $\phi ,\psi$-maxima are predicted with high fidelity, i.e. protein conformers are correctly identified. The central finding of this study is that within the same framework, disordered residues are clearly identified as such, and predicted backbone angle distributions inform on intricacies beyond a generic random coil. Therefore, this study poses a major milestone towards resolving the long-lasting challenge (Bolik-Coulon et al. 2019, p. 72) to interpret CCR applied to disordered proteins, and thus to experimentally determine structural propensities in disordered proteins and regions.

We found that $\tau _{C',C'C_{\alpha }}$ is an optimal dynamical proxy for remote CCR rates. Besides omitting fast librational motions that are exclusive to the $NH^N$ bond, the $C'$-CSA tensor and $C'C_{\alpha }$ bond probe multiple spatial directions (as opposed to effectively $NH^N$-based relaxation, see Sect. “Identification of optimal dynamical proxy”). Therefore, $\tau _{C',C'C_{\alpha }}$ is more representative of the overall local dynamics of the residue, which also governs the dynamics of remote CCR rates in the protein backbone. For rates which involve $H^N$, thus comprising its characteristic dynamics to a certain degree, it makes sense not completely eliminating $NH^N$-based relaxation from the dynamical proxy, for example by combining $\tau _{C',C'C_{\alpha }}$ and $\tau _{N,NH^N}$. This is expected to be beneficial especially in a disordered context, where the peptide plane experiences higher and anisotropic motility (Ceccolini et al. 2024).

In future studies, predictions of protein and in particular IDP ensembles on the basis of CCR could be further improved by employing a more sophisticated prior (e.g., residue-type-specific prior distributions, or even protein-specific ensembles derived from MD), a predictor of higher dimensionality (e.g., a machine learning algorithm like a neural network) and inclusion of additional observables that hold complementary information (e.g., novel CCR rates, chemical shifts of different nuclei, scalar couplings, residual dipolar couplings, etc.).

Besides methodological improvements, it will be very exciting to see future applications of this method in an experimental context to disordered proteins and regions, revealing their structure propensities and shedding light on their diverse and context-dependent biological roles.

## Supplementary Information

Below is the link to the electronic supplementary material.

Supplementary file 1 (pdf 2491 KB)

## Appendix

According to Eq. (2), which relates the integral of the correlation function *C*(*t*) to the zero-frequency spectral density function *J*(0), and Eqs. (9)–(11), the expressions for specific CCR rates are obtained as:

17 $$\begin{aligned} & \Gamma _{N_i H_i^N,C_i^{\alpha }H_i^{\alpha }} = \frac{2}{5}\bigg (\frac{\mu _0\hbar }{4\pi }\bigg )^2 \gamma _C \gamma _N \gamma _H^2 J_{C_i^{\alpha }H_i^{\alpha },N_i H_i^N}(0) \end{aligned}$$

18 $$\begin{aligned} & \Gamma _{C_{i-1}^{'},C_i^{\alpha }H_i^{\alpha }} = \frac{4}{15} \gamma _C B_0 \frac{\mu _0\hbar }{4\pi } \gamma _C \gamma _H \sum _{k=x,y} (\sigma ^{C'}_{kk}-\sigma ^{C'}_{zz}) J_{C_i^{\alpha }H_i^{\alpha },kk^{C'}_{i-1}}(0) \end{aligned}$$

19 $$\begin{aligned} & \Gamma _{C_i^{\alpha }H_i^{\alpha },N_{i+1} H_{i+1}^N} = \frac{2}{5} \bigg (\frac{\mu _0\hbar }{4\pi }\bigg )^2 \gamma _C \gamma _N \gamma _H^2 J_{C_i^{\alpha }H_i^{\alpha },N_{i+1} H_{i+1}^N}(0) \end{aligned}$$

20 $$\begin{aligned} & \Gamma _{C_i^{\alpha }H_i^{\alpha },C_{i}^{'}} = \frac{4}{15} \gamma _C B_0 \frac{\mu _0\hbar }{4\pi } \gamma _C \gamma _H \sum _{k=x,y} (\sigma ^{C'}_{kk}-\sigma ^{C'}_{zz}) J_{C_i^{\alpha }H_i^{\alpha },kk^{C'}_{i}}(0) \end{aligned}$$

21 $$\begin{aligned} & \Gamma _{C_{i}^{'},H_i^{\alpha }H_{i+1}^{N}} = \frac{4}{15} \gamma _C B_0 \frac{\mu _0\hbar }{4\pi } \gamma _H^2 \sum _{k=x,y} (\sigma ^{C'}_{kk}-\sigma ^{C'}_{zz}) J_{H_i^{\alpha }H_{i+1}^{N},kk^{C'}_{i}}(0) \end{aligned}$$

22 $$\begin{aligned} & \Gamma _{N_i H_i^{N},N_{i+1} H_{i+1}^N} = \frac{2}{5}\bigg (\frac{\mu _0\hbar }{4\pi }\bigg )^2 \gamma _N^2 \gamma _H^2 J_{N_i H_i^{N},N_{i+1} H_{i+1}^N}(0) \end{aligned}$$

23 $$\begin{aligned} & \Gamma _{C_{i-1}^{'},C_{i}^{'}} = \frac{8}{45} \gamma _C^2 B_0^2 \sum _{\begin{array}{c} k=x,y \\ l=x,y \end{array}} (\sigma ^{C'}_{kk}-\sigma ^{C'}_{zz})(\sigma ^{C'}_{ll}-\sigma ^{C'}_{zz}) J_{kk^{C'}_{i},ll^{C'}_{i-1}}(0) \end{aligned}$$

24 $$\begin{aligned} & \Gamma _{N_i H_i^{N},C_i^{'}} = \frac{4}{15} \gamma _C B_0 \frac{\mu _0\hbar }{4\pi } \gamma _N \gamma _H \sum _{k=x,y} (\sigma ^{C'}_{kk}-\sigma ^{C'}_{zz}) J_{N_i H_i^{N},kk^{C'}_i}(0) \end{aligned}$$

25 $$\begin{aligned} & \Gamma _{N_{i},N_{i}H^{N}_{i}} = \frac{4}{15} \gamma _N B_0 \frac{\mu _0\hbar }{4\pi } \gamma _N \gamma _H (\sigma ^N_{xx}-\sigma ^N_{zz}) J_{N_i H_i^N,xx^N_{i}}(0) \end{aligned}$$

26 $$\begin{aligned} & \Gamma _{C_{i}^{'},C^{'}_{i}C^{\alpha }_{i}} = \frac{4}{15} \gamma _C B_0 \frac{\mu _0\hbar }{4\pi } \gamma _C^{2} \sum _{k=x,y} (\sigma ^{C'}_{kk}-\sigma ^{C'}_{zz}) J_{C^{'}_iC_i^{\alpha },kk_{i}^{C'}}(0) \end{aligned}$$

Note that expressions $\Gamma$ in Eqs. (25) and (26) are experimentally obtained by a linear combination of transversal and longitudinal rates in the form of $\Gamma =\Gamma _{xy}-0.5 \,\Gamma _z$ (Kroenke et al. 1998; Pelupessy et al. 2003; Kadeřávek et al. 2014; Kauffmann et al. 2021)
