# Supplementary material for: Local structure propensities in disordered proteins from cross-correlated NMR spin relaxation
Source: J Biomol NMR. 2025 Feb 26;79(2):115–27. doi: 10.1007/s10858-025-00460-3 (PMC12078414; doi:10.1007/s10858-025-00460-3)
Supplement: Supplementary file 1 — (pdf 2491 KB) [file 10858_2025_460_MOESM1_ESM.pdf]

Supplementary Information for "Local structure propensities in disordered proteins from cross-correlated NMR spin relaxation"

Daniel Braun[1*†], Clemens Kauffmann[1†], Andreas Beier[1], Irene Ceccolini[1], Olga O. Lebedenko[2], Nikolai R. Skrynnikov[2,3] and Robert Konrat[1*]

[1*]Department of Computational and Structural Biology, University of Vienna, Campus Vienna Biocenter 5, Vienna, 1030, Vienna, Austria.
[2]Laboratory of Biomolecular NMR, St. Petersburg State University, St. Petersburg, Russia.
[3]Department of Chemistry, Purdue University, West Lafayette, Indiana,USA.

*Corresponding author(s). E-mail(s): daniel.braun@univie.ac.at; robert.konrat@univie.ac.at;
†These authors contributed equally to this work.

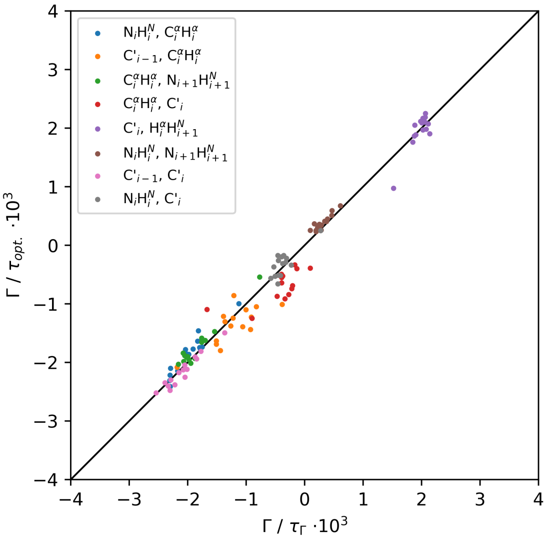

Fig. 1: NH4: rates' structural component $A_\Gamma = \Gamma/\tau_\Gamma$ vs. approximated structural component Γ/τ_{opt}, where $\tau_{opt} = 0.7 \cdot \tau_{C',C'C^\alpha} + 0.3 \cdot \tau_{N,NH^N}$ for all rates containing the backbone-H^N and $\tau_{opt} = \tau_{C',C'C^\alpha}$ for the remaining rates. The distribution has an $R^2 = 0.99$ and the slope of a linear fit through $(0,0)$ is 0.99.

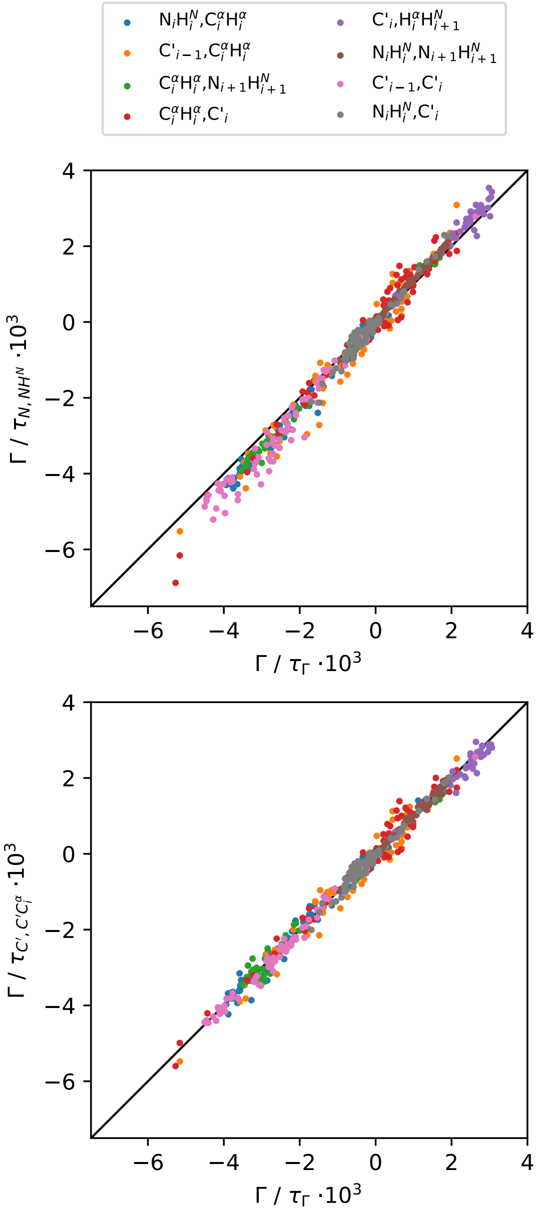

Fig. 2: UBQ: rates' structural component $A_\Gamma = \Gamma/\tau_\Gamma$ vs. approximated structural component a) $\Gamma/\tau_{N,NH^N}$ and b) $\Gamma/\tau_{C',C'C^\alpha}$. Both distributions have an $R^2 = 0.99$. The slope of a linear fit through $(0, 0)$ is 1.13 for a) and 1.00 for b).

Table 1: The model protein backbone in x,y,z-coordinates with $\phi = \psi = 180°$

Atom	x [Å]	y [Å]	z [Å]
C^{α}_{i-1}	3.68942000	-0.08826000	-1.01622000
C'_{i-1}	2.16413000	-0.09939000	-1.13575000
O_{i-1}	1.63267000	-0.19660000	-2.24073000
N_i	1.46000000	0.00000000	0.00000000
H^N_i	1.87649660	0.00860880	0.93105600
C^{α}_i	0.00000000	0.00000000	0.00000000
H^{α}_i	-0.27469536	0.89749108	-0.58883552
C'_i	-0.54830000	0.12453000	1.42294000
O_i	0.22409000	0.20798000	2.37653000
C^{β}_i	-0.53833000	-1.26272000	-0.67575000
N_{i+1}	-1.88047000	0.13714000	1.56711000
H^N_{i+1}	-2.53918600	0.13894540	0.78834000

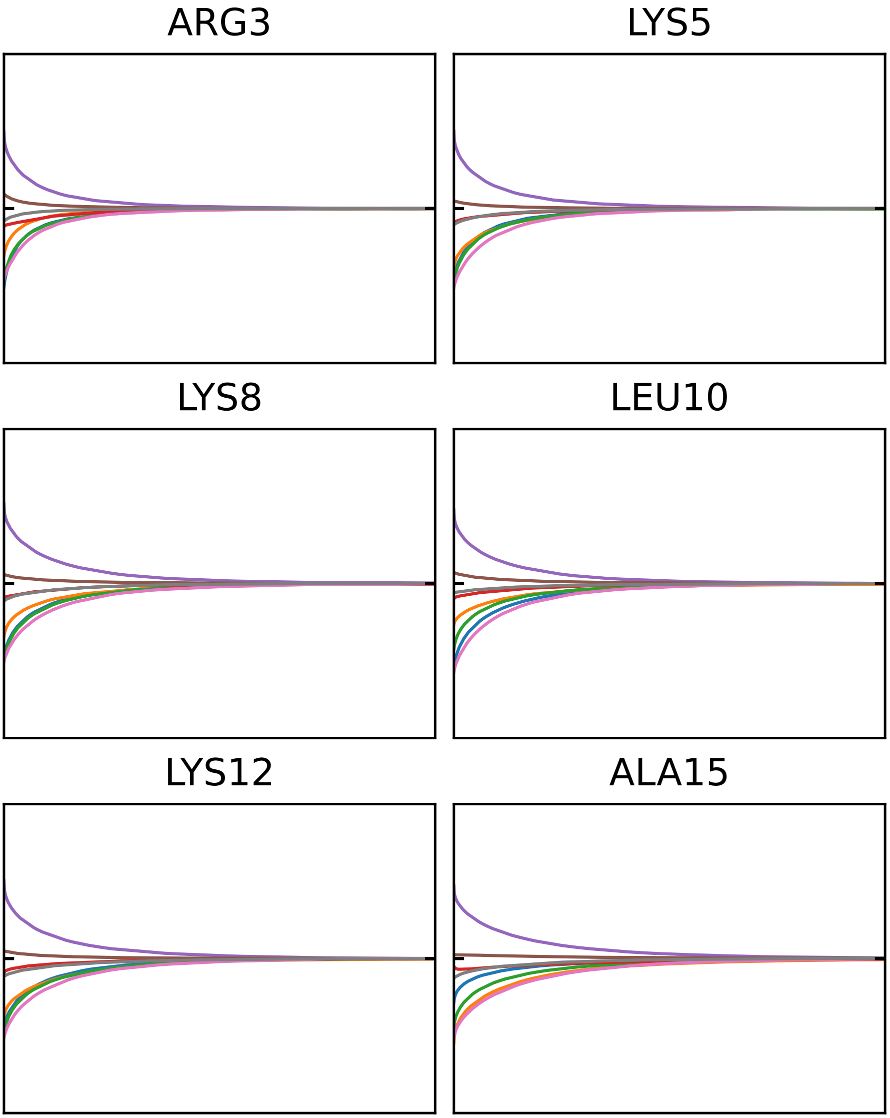

Fig. 3: NH4 correlation functions $C(t)$, full caption, see below.

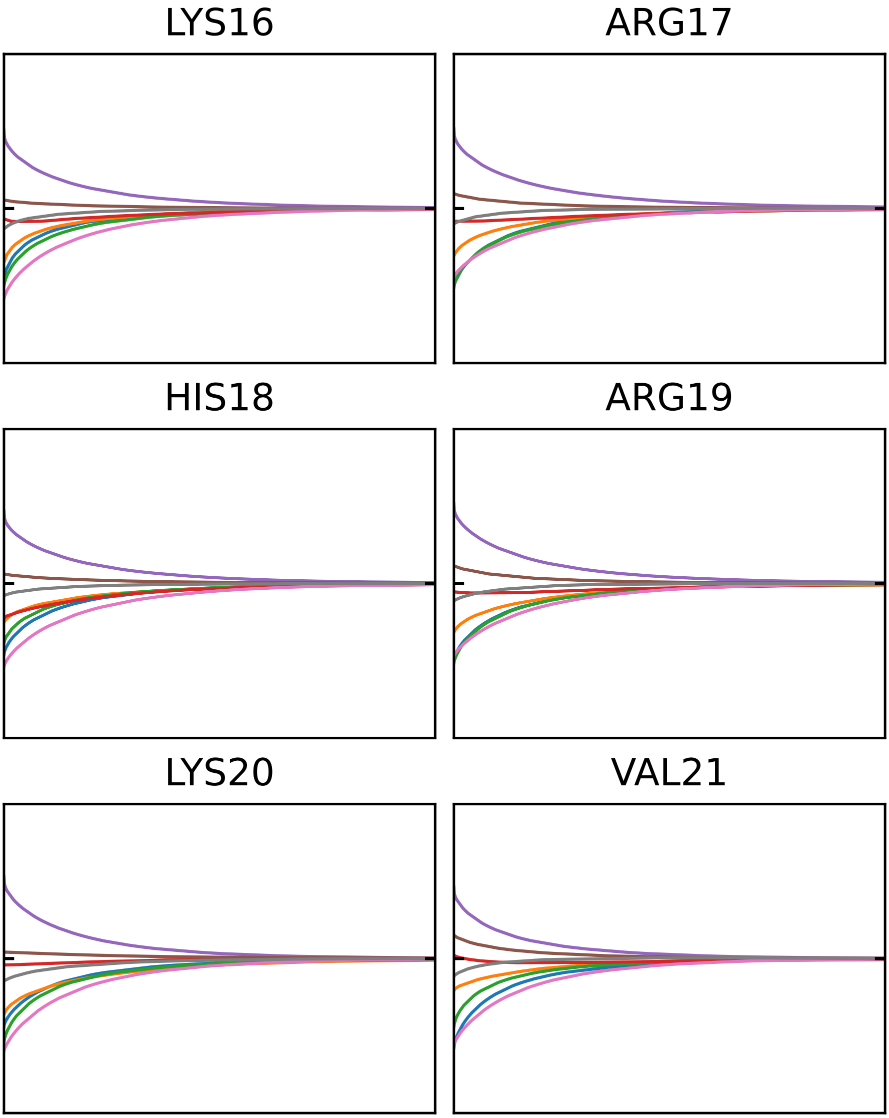

Fig. 3: NH4 correlation functions $C(t)$, full caption, see below.

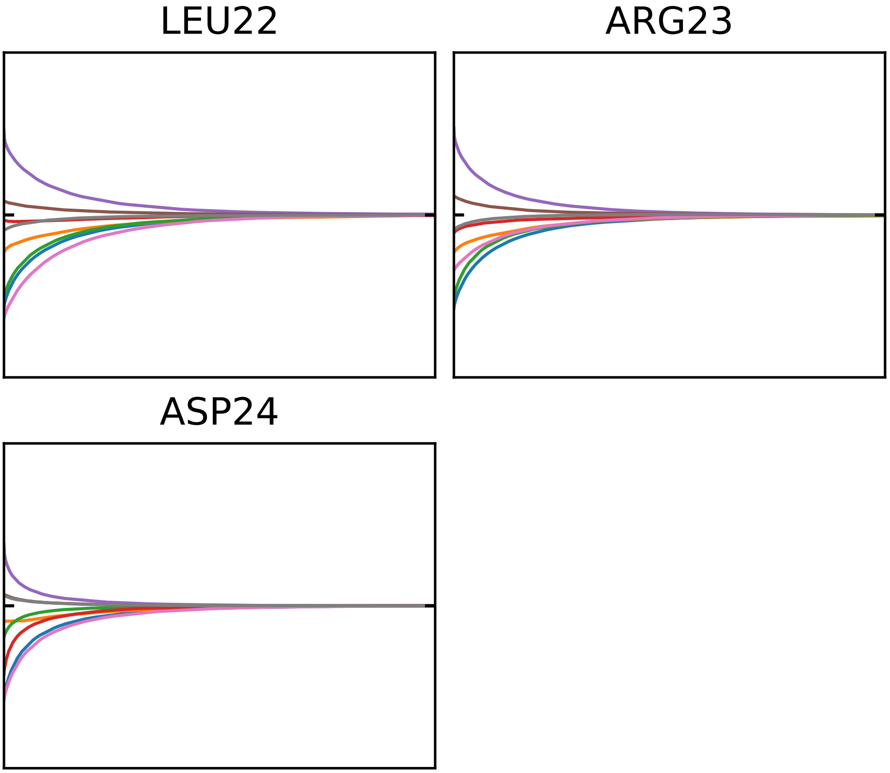

Fig. 3: NH4 time correlation functions $C(t)$. The x-axis extends from $t = 0$ ps to $t = 10000$ ps; the y-axis from -0.004 to 0.004 s^{-2} and TCFs have the amplitude A_Γ as defined in the Methods section. The color code for each rate is consistent with the rest of the paper, see Fig. S1. Note that all correlation functions converge well towards 0 within the shown time interval, allowing for numerical integration when calculating rates Γ.

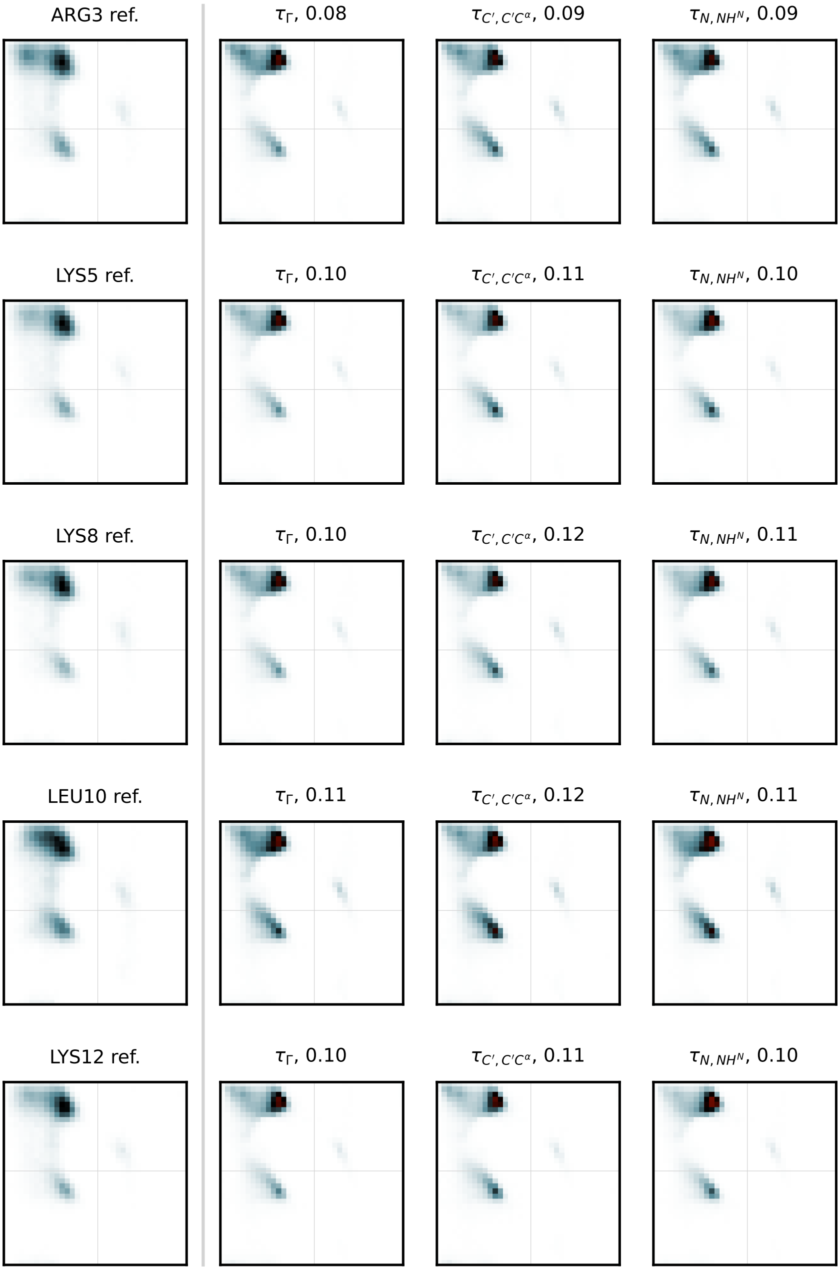

Fig. 4: NH4 ϕ,ψ-distributions, full caption, see below.

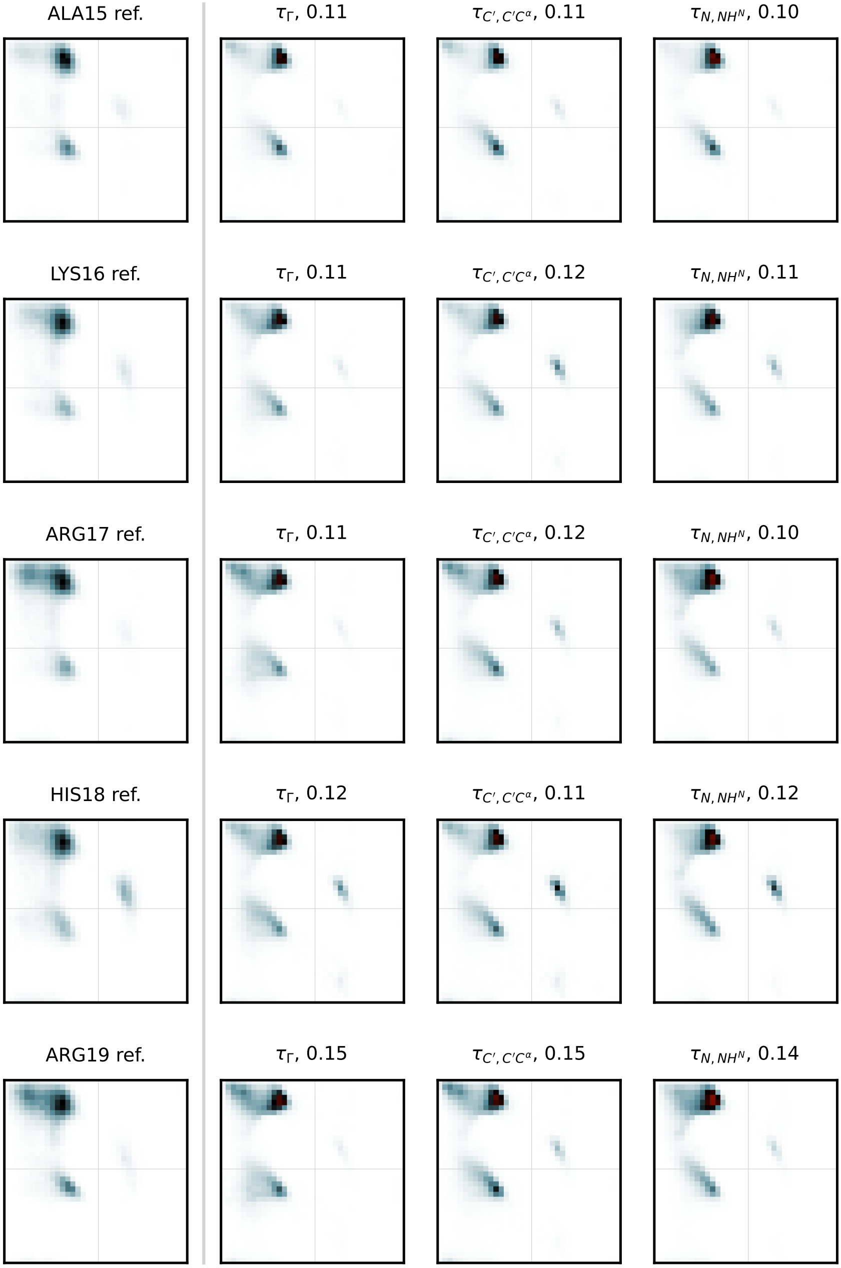

Fig. 4: NH4 ϕ,ψ-distributions, full caption, see below.

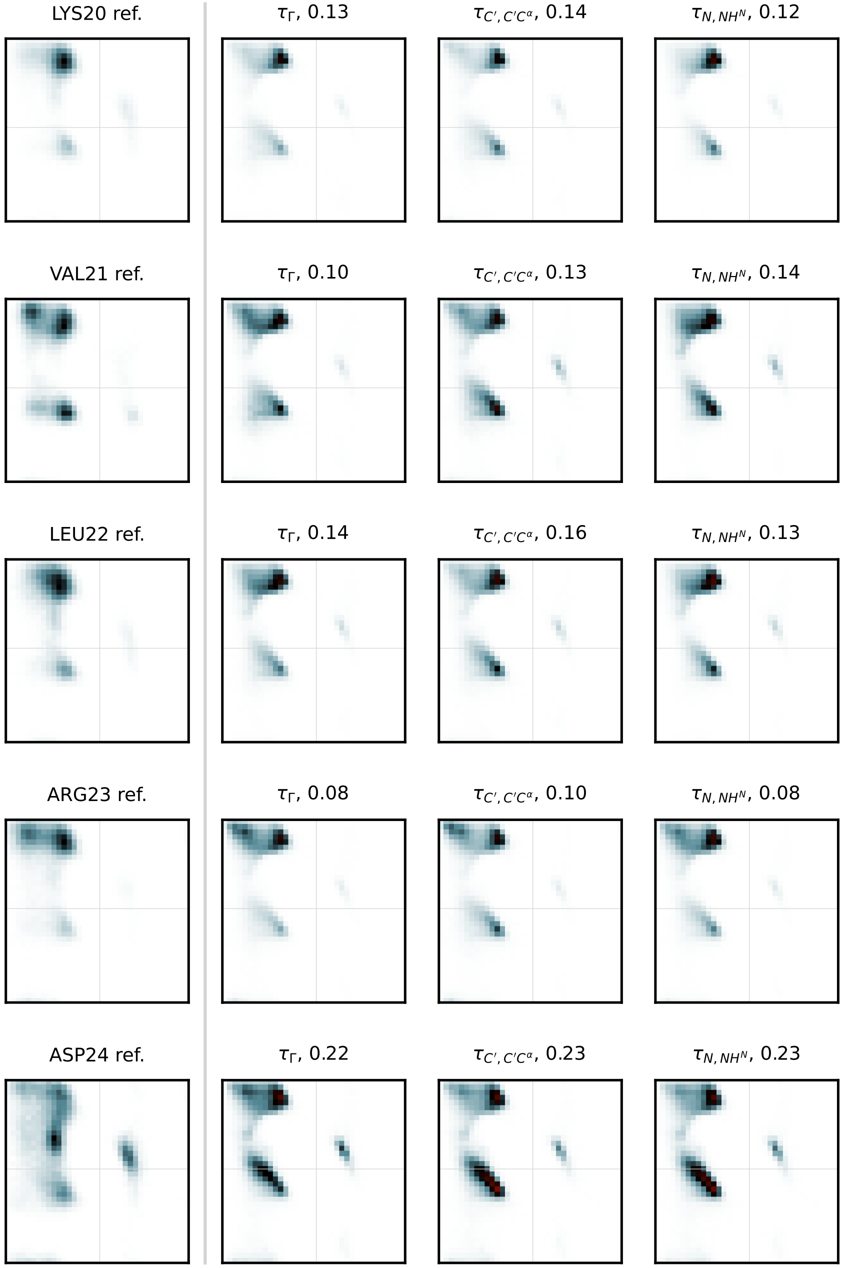

Fig. 4: NH4 ϕ,ψ-distributions, full caption, see below.

Fig. 4: NH4 ϕ,ψ-distributions. Each line represents a residue. From left to right: reference distribution $\boldsymbol{r}_{\phi,\psi}$ extracted directly from simulation; distribution predicted from rates together with the rates' own correlation time τ_{Γ}; with $\tau_{C',C'C^{\alpha}}$; with $\tau_{N,NH^{N}}$. For each residue, the range of the color scale is defined by the range of $\boldsymbol{r}_{\phi,\psi}$ (see main paper). For all predictions the Jensen-Shannon divergence with respect to $\boldsymbol{r}_{\phi,\psi}$ is provided (see Methods). The mean JS divergence over all residues is ~0.12 for τ^{Γ}, ~0.13 for $\tau^{C',C'C^{\alpha}}$ and ~0.12 for $\tau^{N,NH^{N}}$.

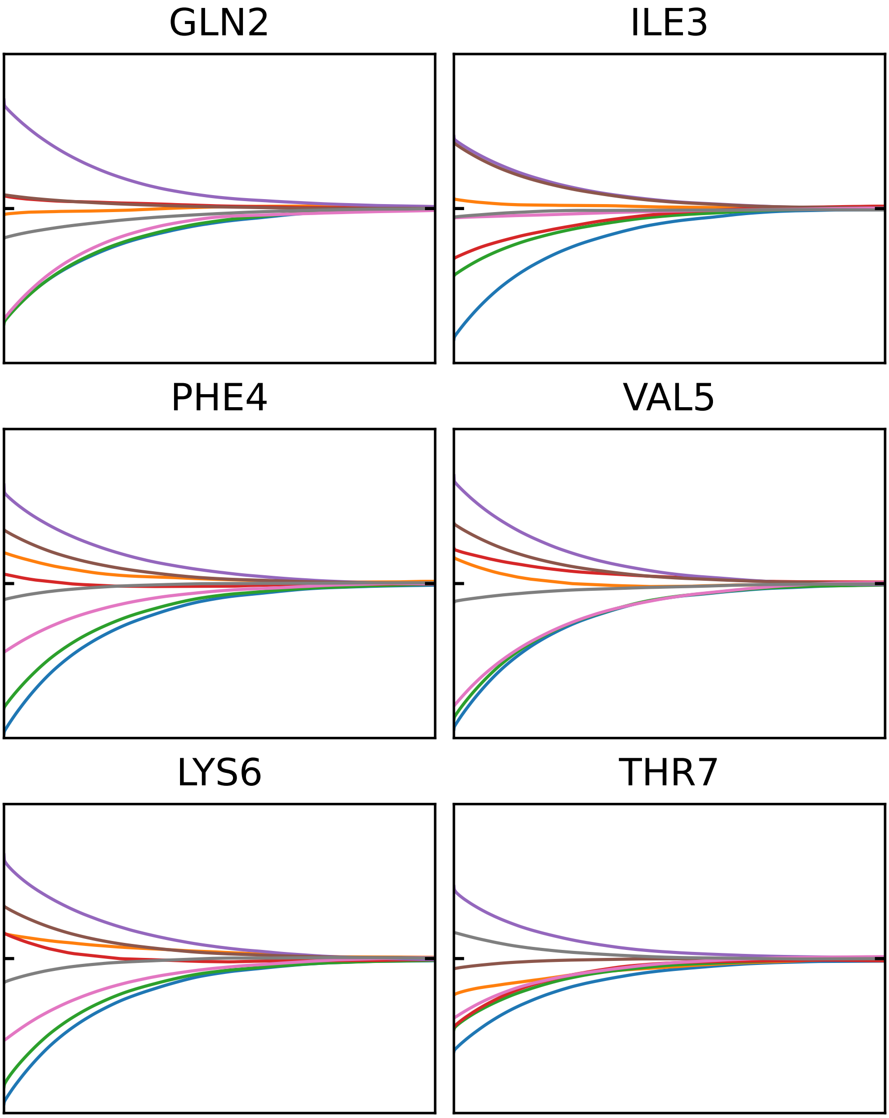

Fig. 5: UBQ correlation functions $C(t)$, full caption, see below.

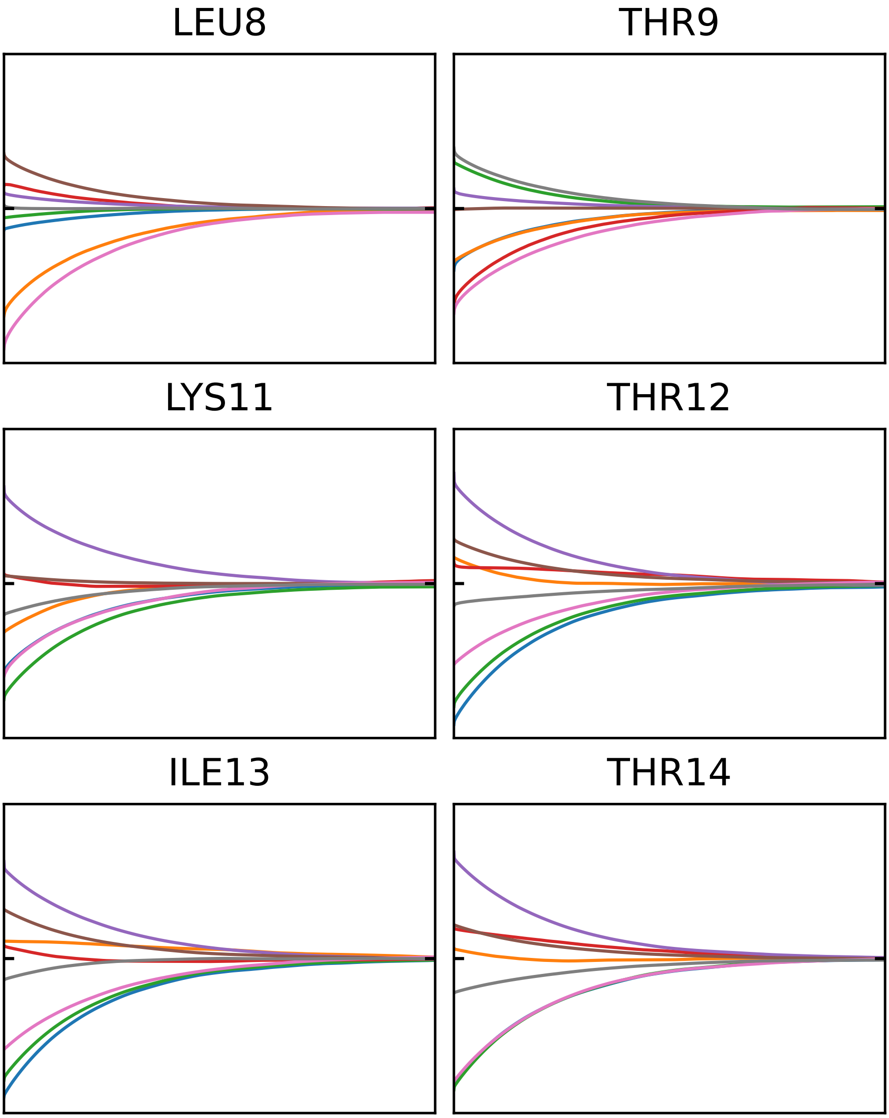

Fig. 5: UBQ correlation functions $C(t)$, full caption, see below.

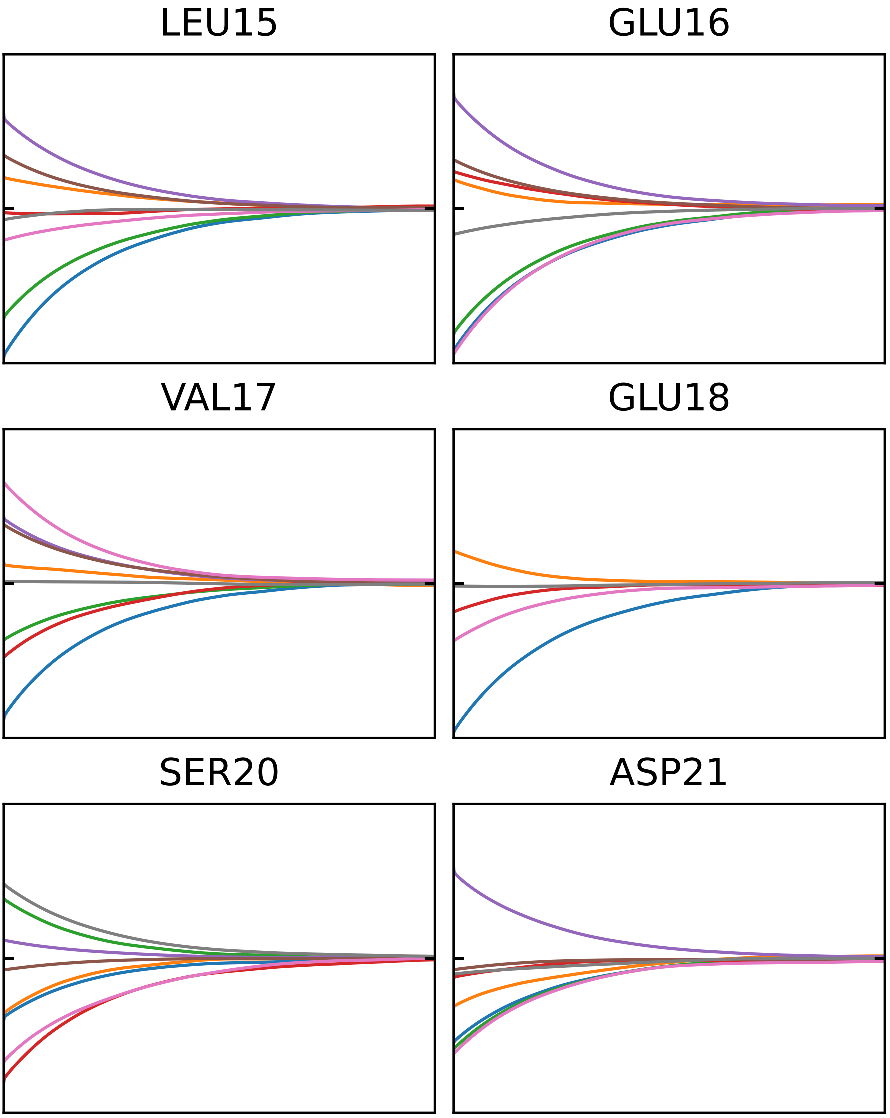

Fig. 5: UBQ correlation functions $C(t)$, full caption, see below.

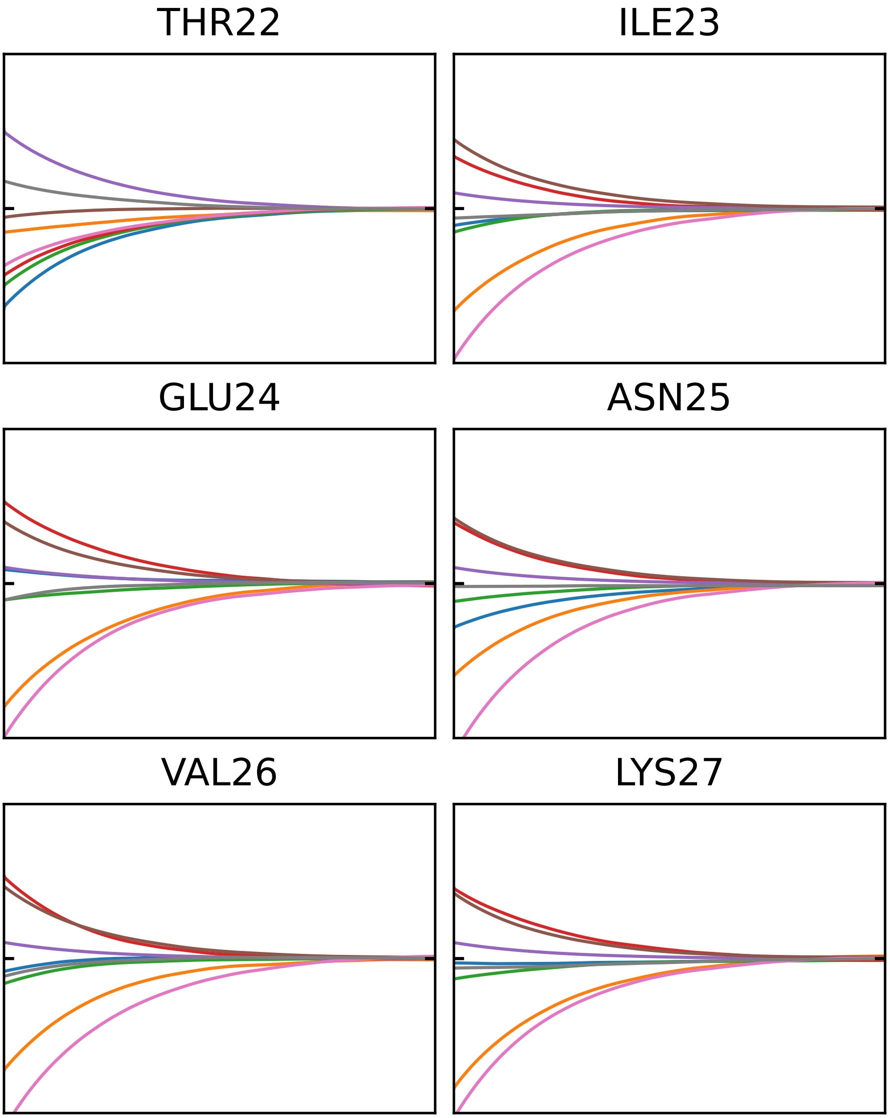

Fig. 5: UBQ correlation functions $C(t)$, full caption, see below.

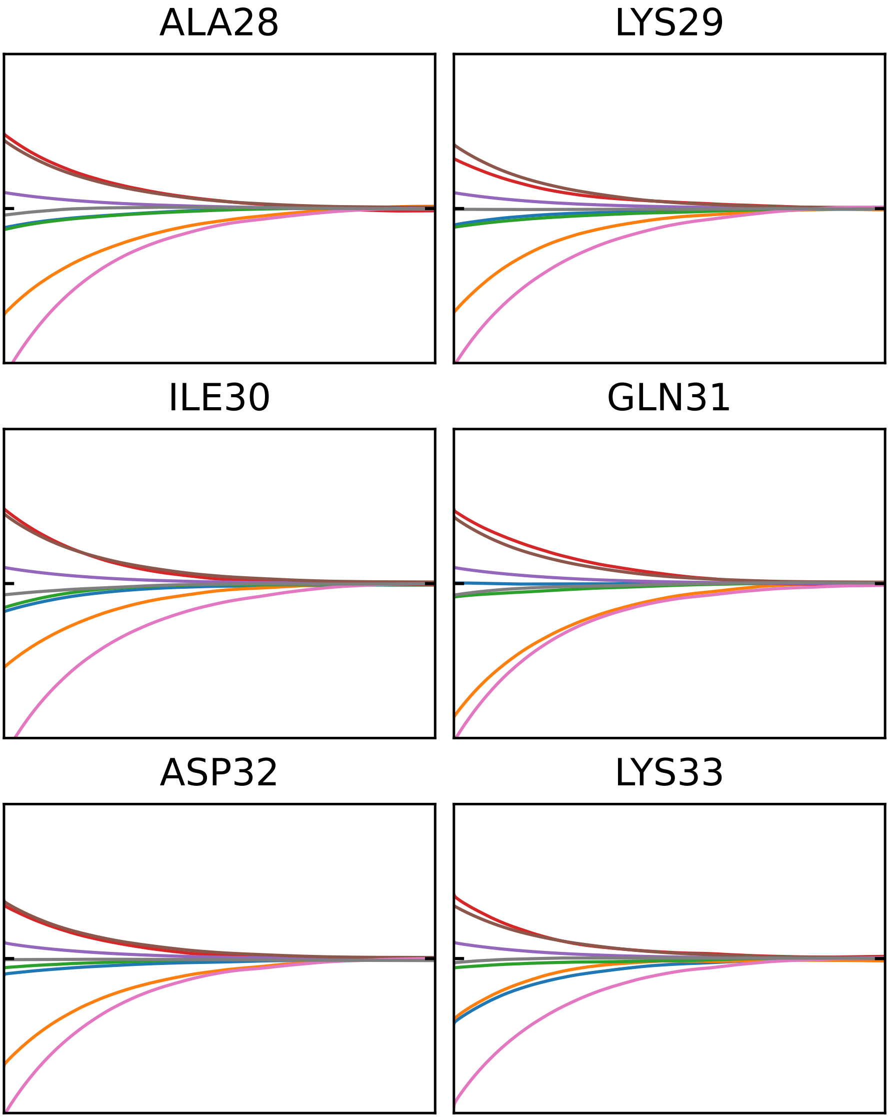

Fig. 5: UBQ correlation functions $C(t)$, full caption, see below.

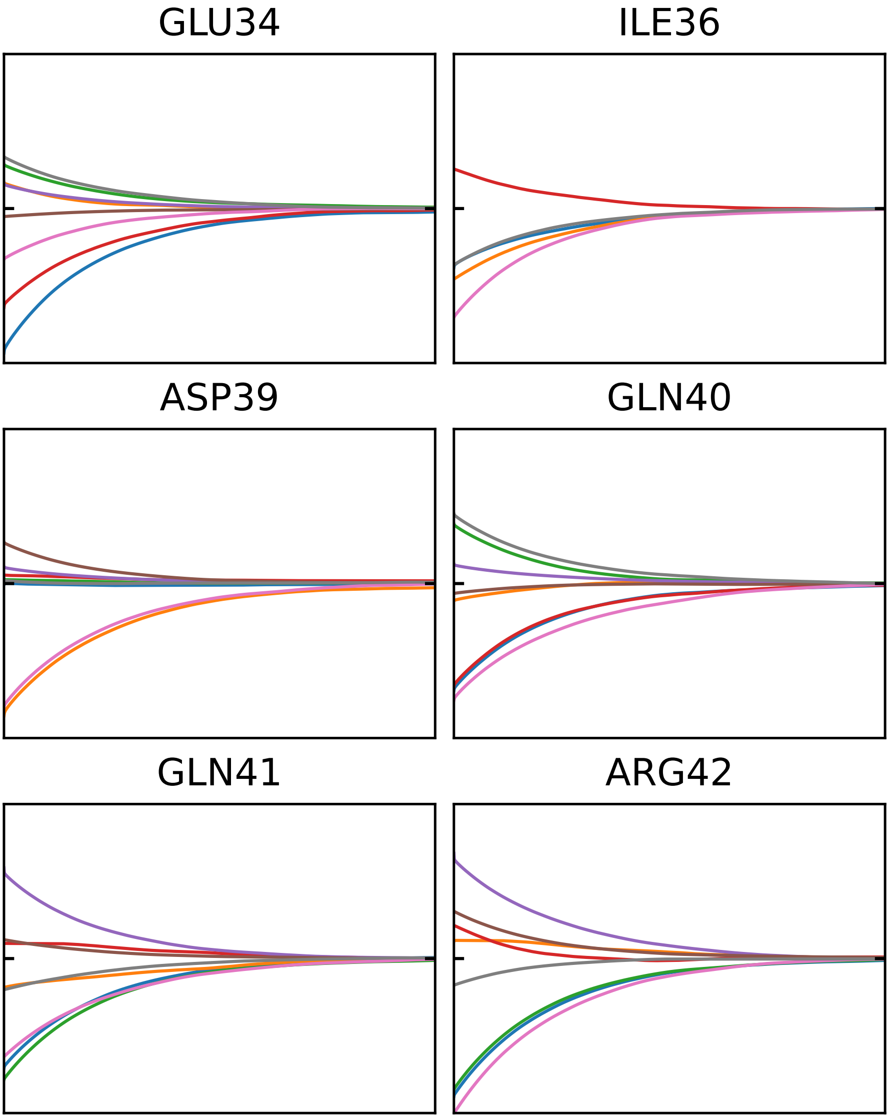

Fig. 5: UBQ correlation functions $C(t)$, full caption, see below.

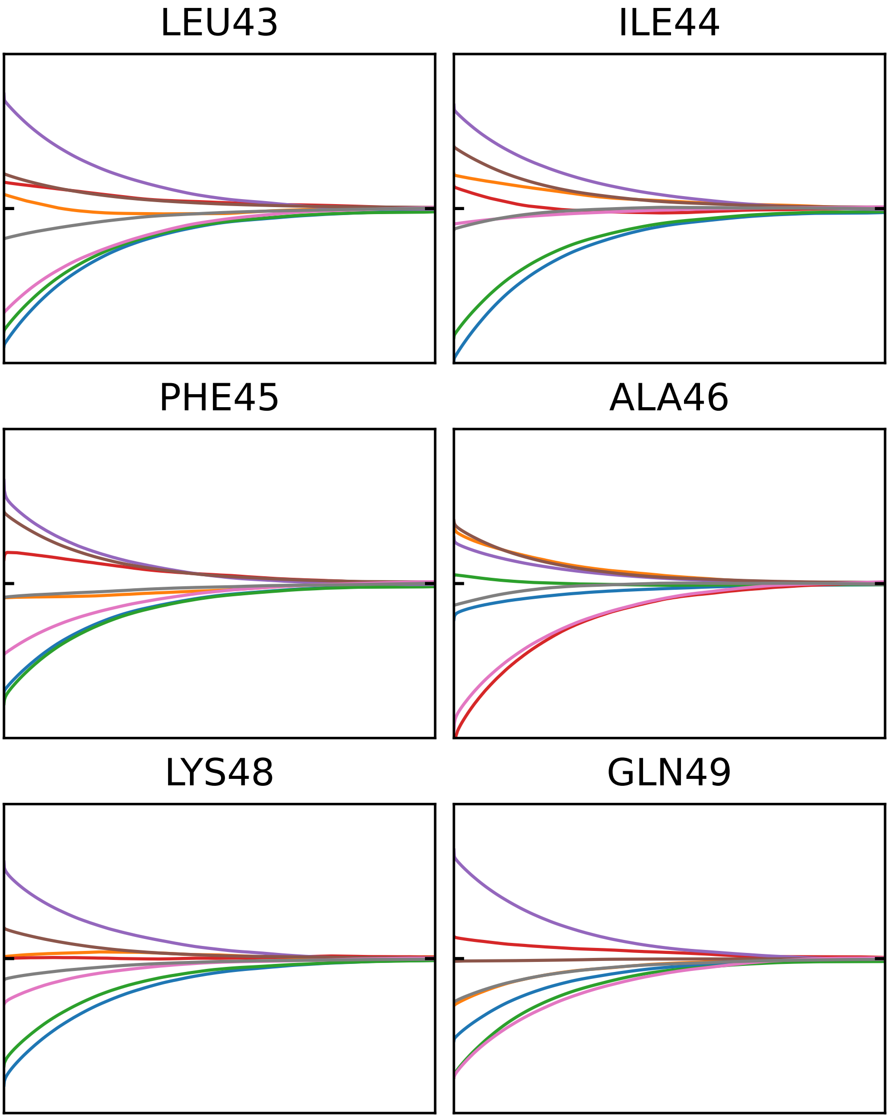

Fig. 5: UBQ correlation functions $C(t)$, full caption, see below.

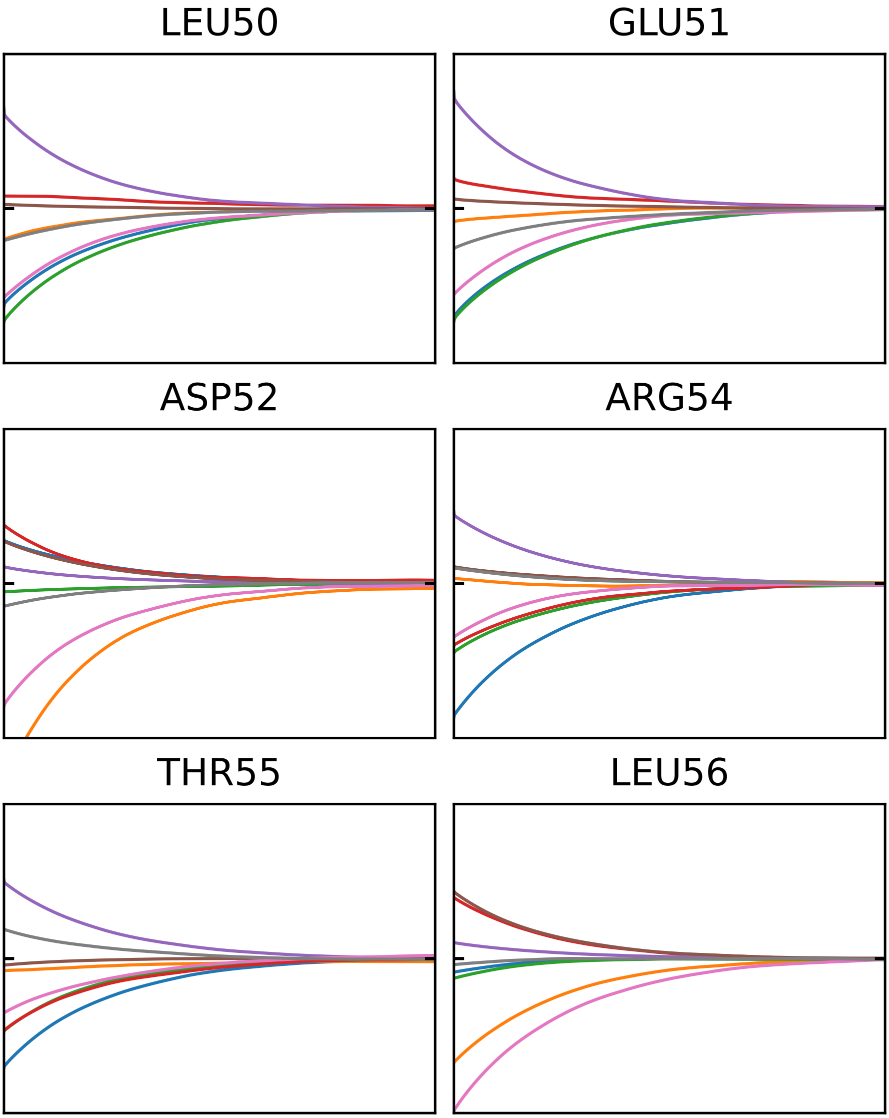

Fig. 5: UBQ correlation functions $C(t)$, full caption, see below.

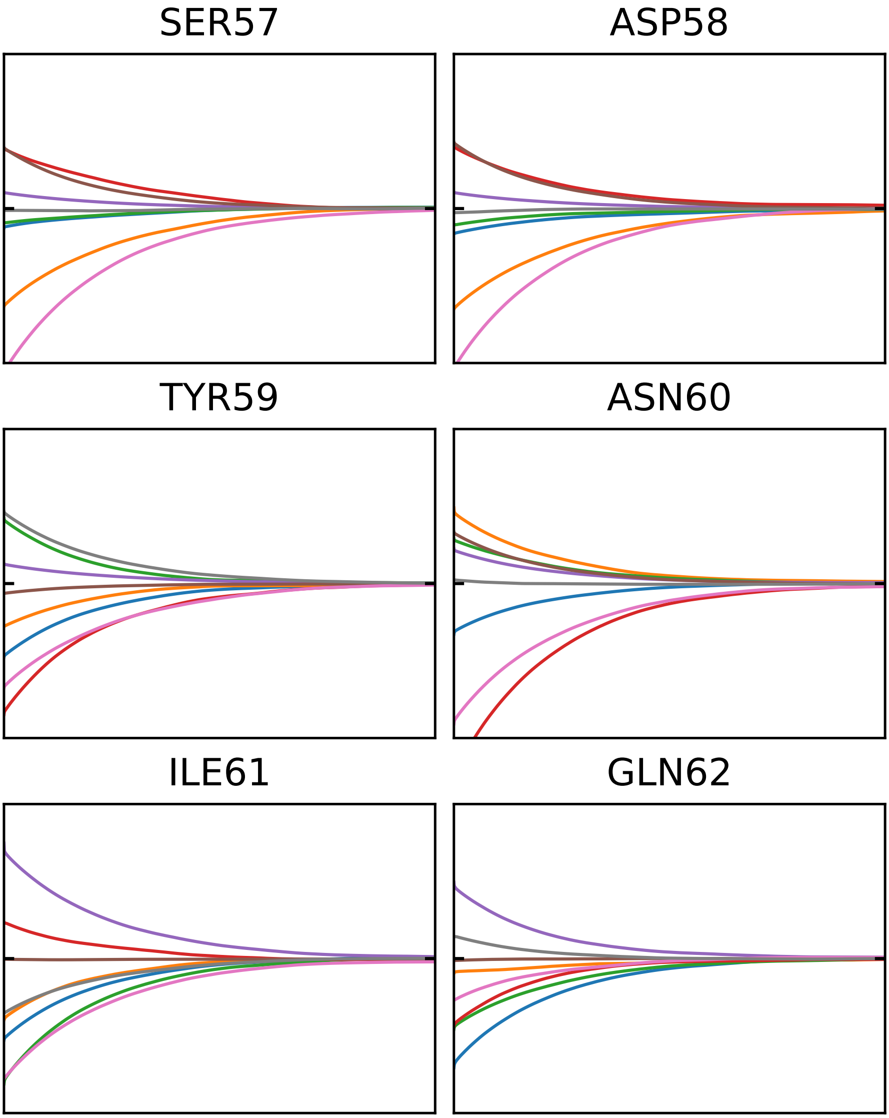

Fig. 5: UBQ correlation functions $C(t)$, full caption, see below.

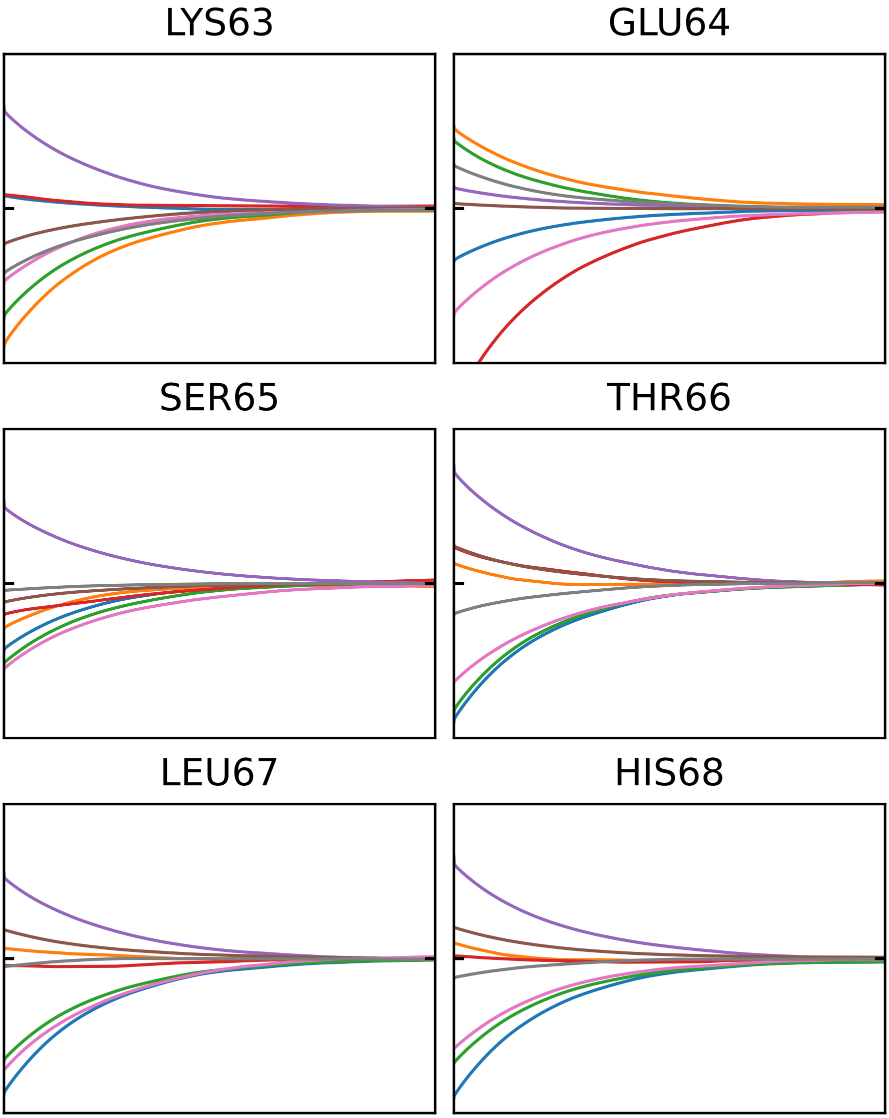

Fig. 5: UBQ correlation functions $C(t)$, full caption, see below.

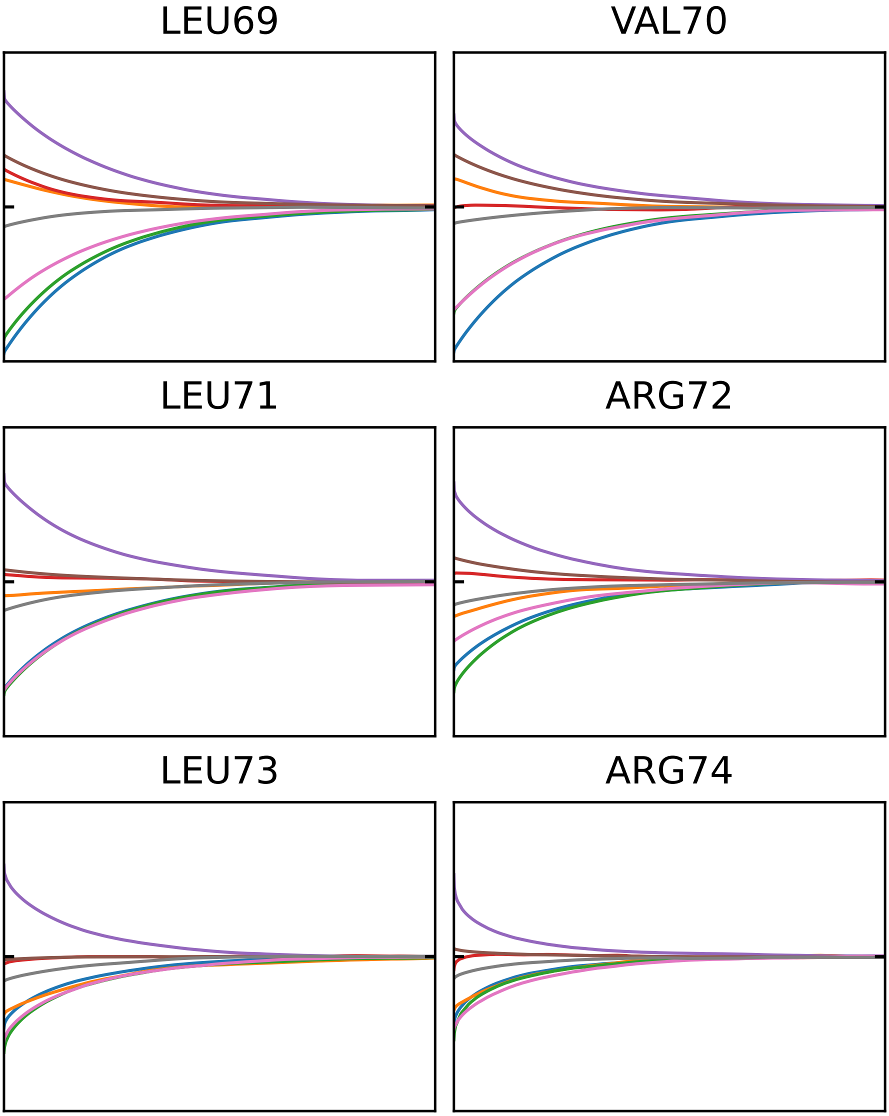

Fig. 5: UBQ time correlation functions $C(t)$. The x-axis extends from $t = 0$ ps to $t = 20000$ ps; the y-axis from -0.004 to 0.004 s^{-2} and TCFs have the amplitude A_Γ as defined in the Methods section. The color code for each rate is consistent with the rest of the paper, see Fig. S1. Note that all correlation functions converge well towards 0 within the shown time interval, allowing for numerical integration when calculating rates Γ.

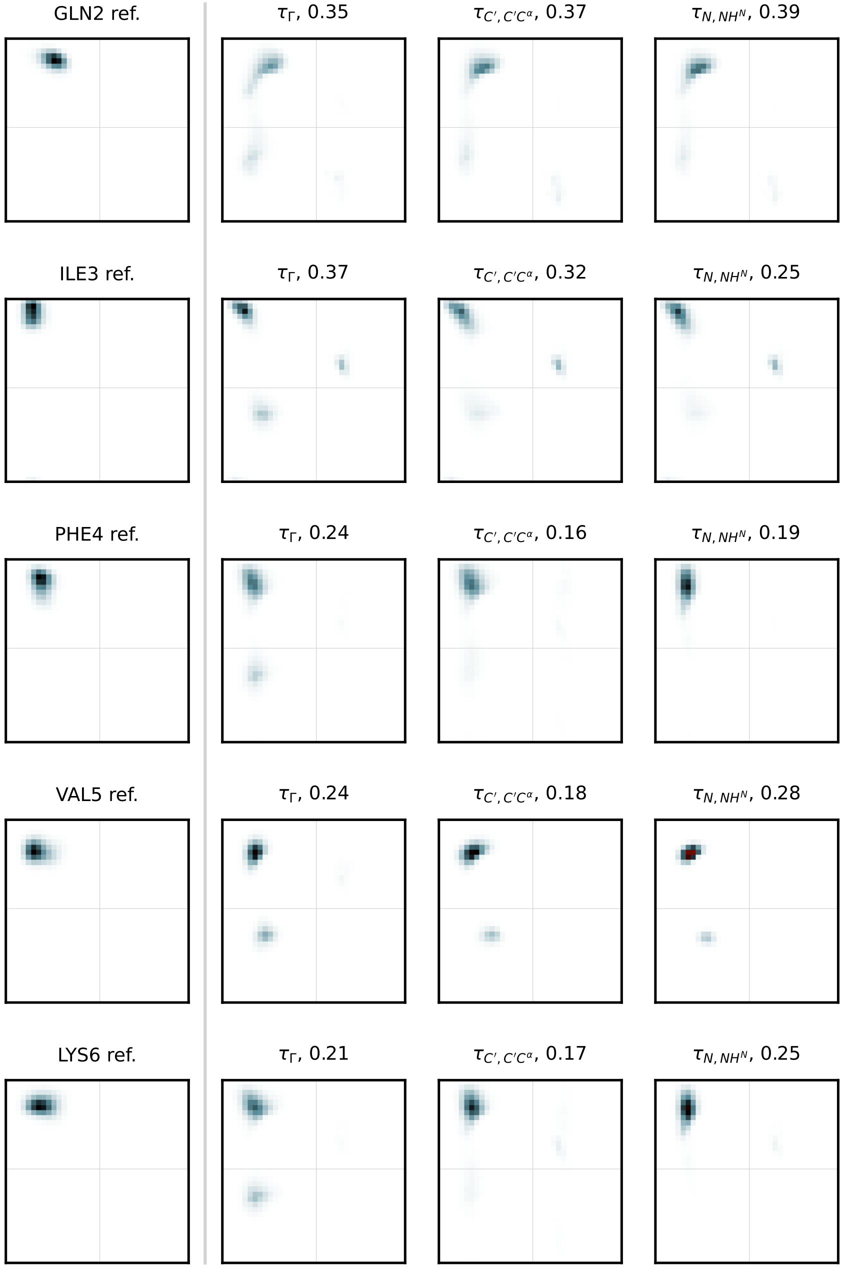

Fig. 6: UBQ ϕ,ψ-distributions, full caption, see below.

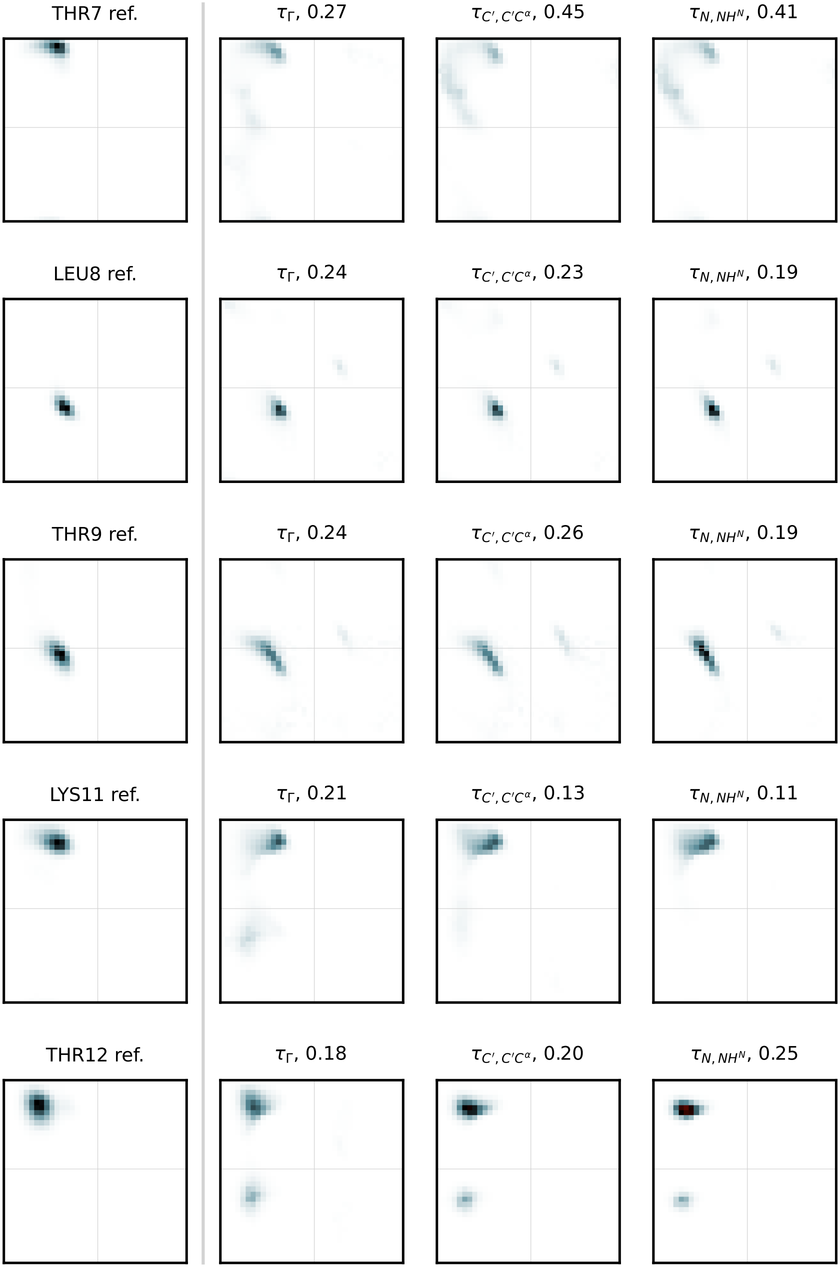

Fig. 6: UBQ ϕ,ψ-distributions, full caption, see below.

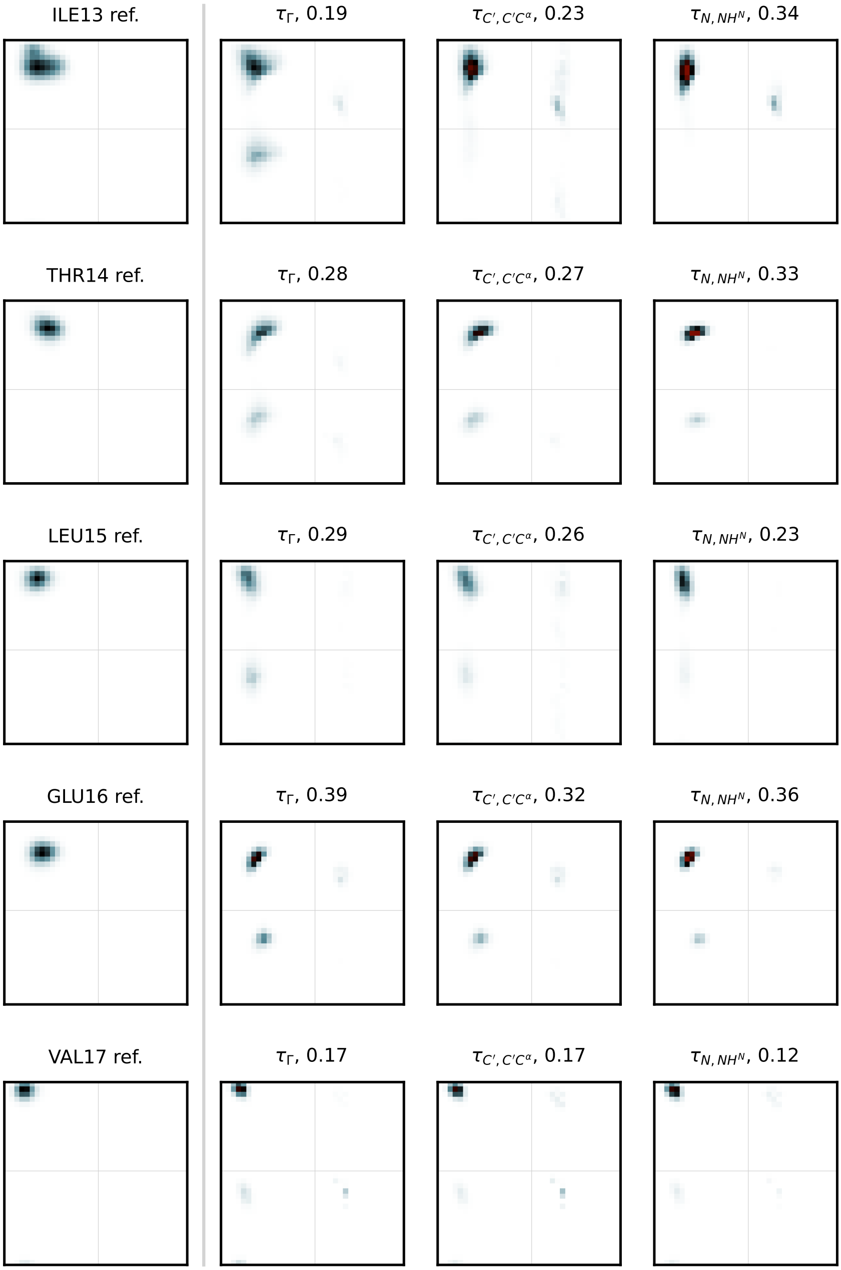

Fig. 6: UBQ ϕ,ψ-distributions, full caption, see below.

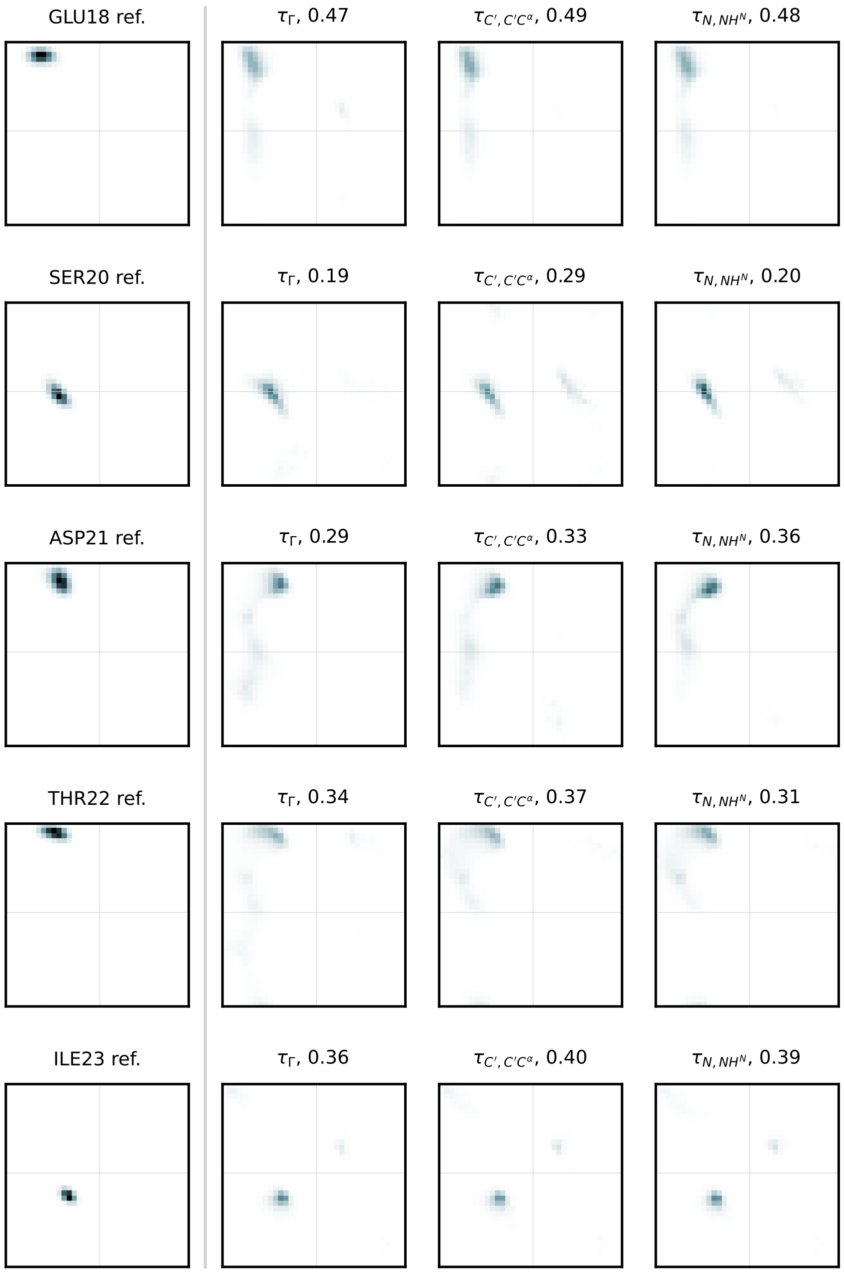

Fig. 6: UBQ ϕ,ψ-distributions, full caption, see below.

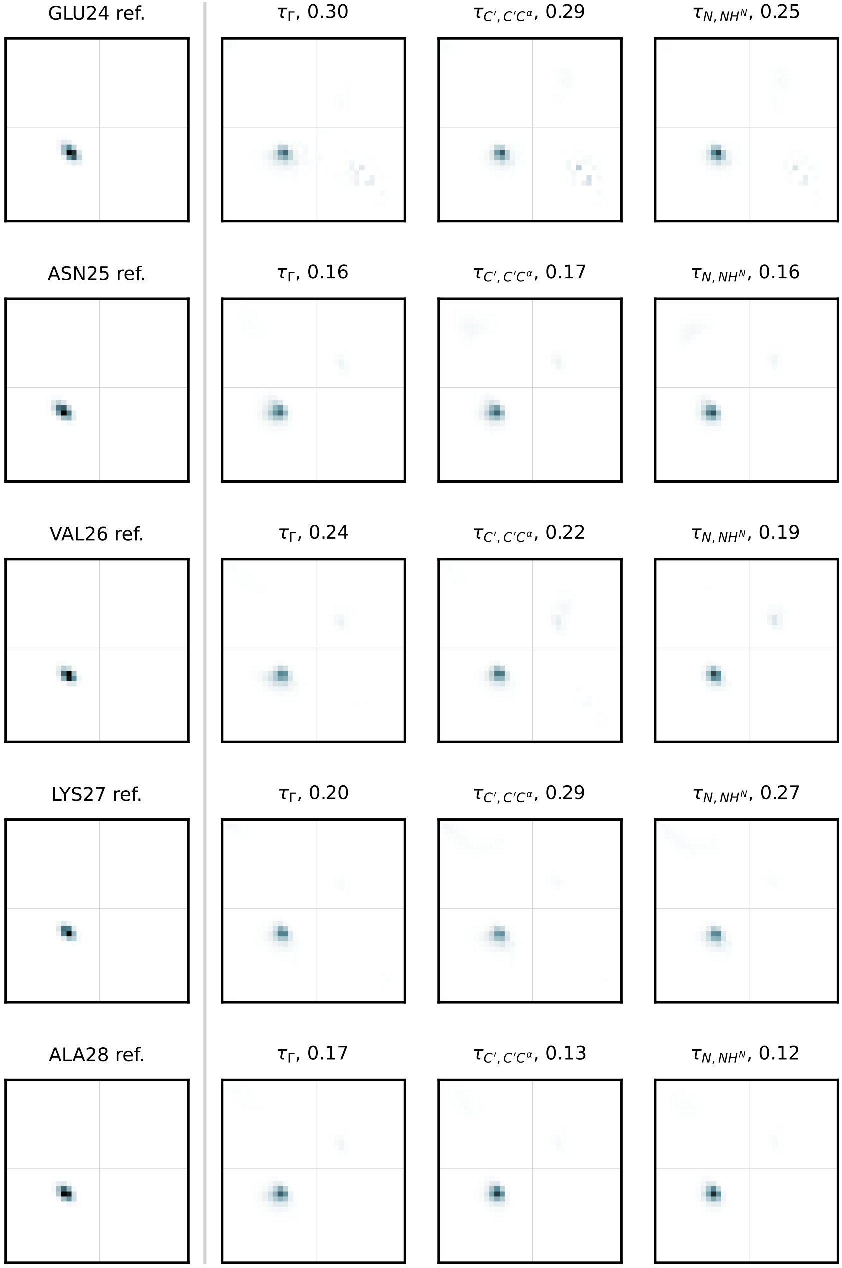

Fig. 6: UBQ ϕ,ψ-distributions, full caption, see below.

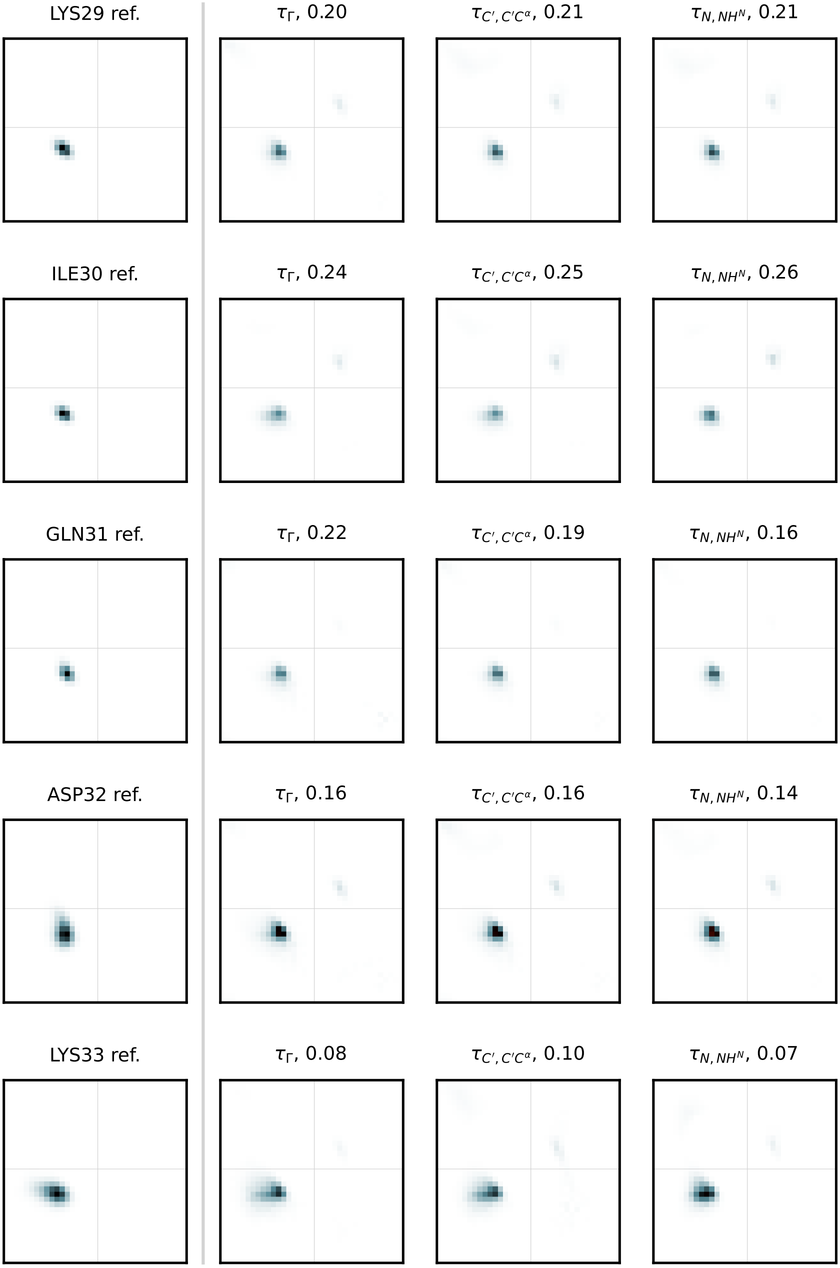

Fig. 6: UBQ ϕ,ψ-distributions, full caption, see below.

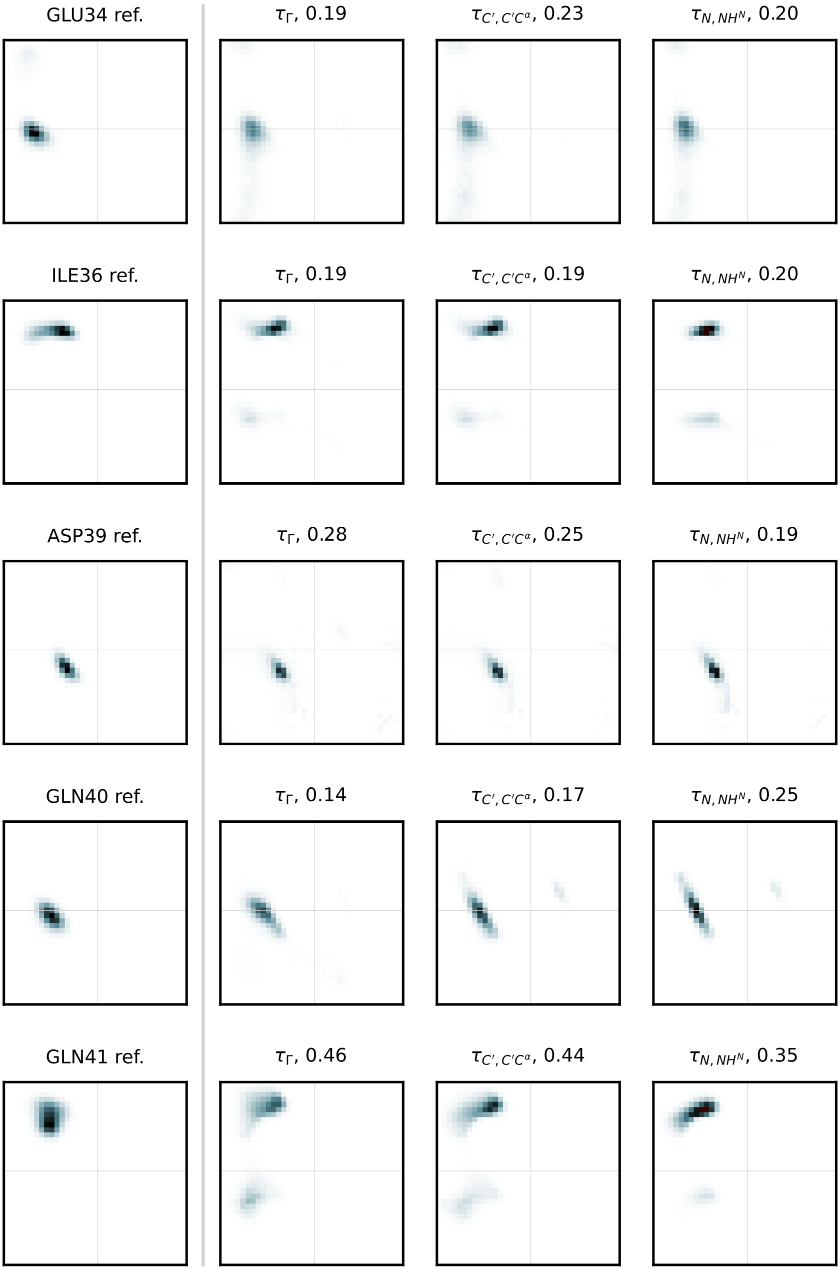

Fig. 6: UBQ ϕ,ψ-distributions, full caption, see below.

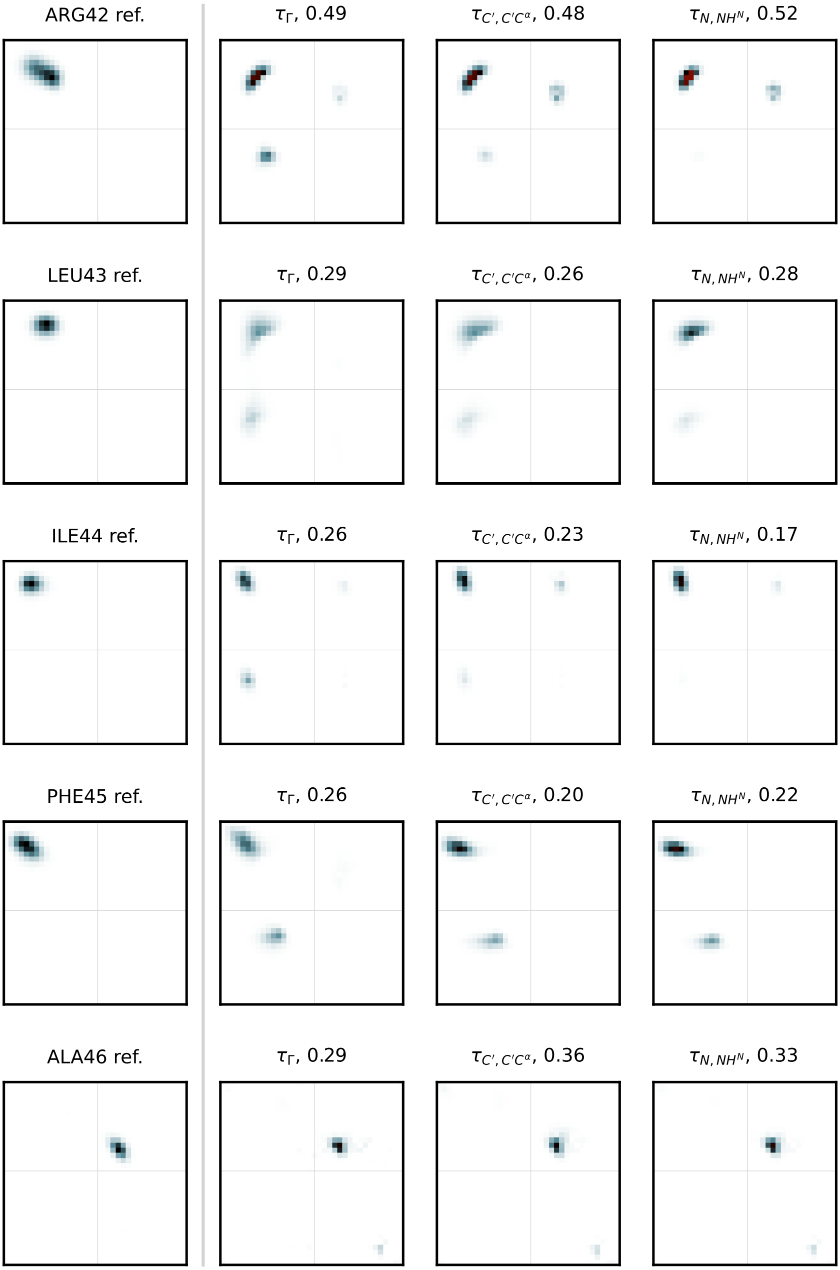

Fig. 6: UBQ ϕ,ψ-distributions, full caption, see below.

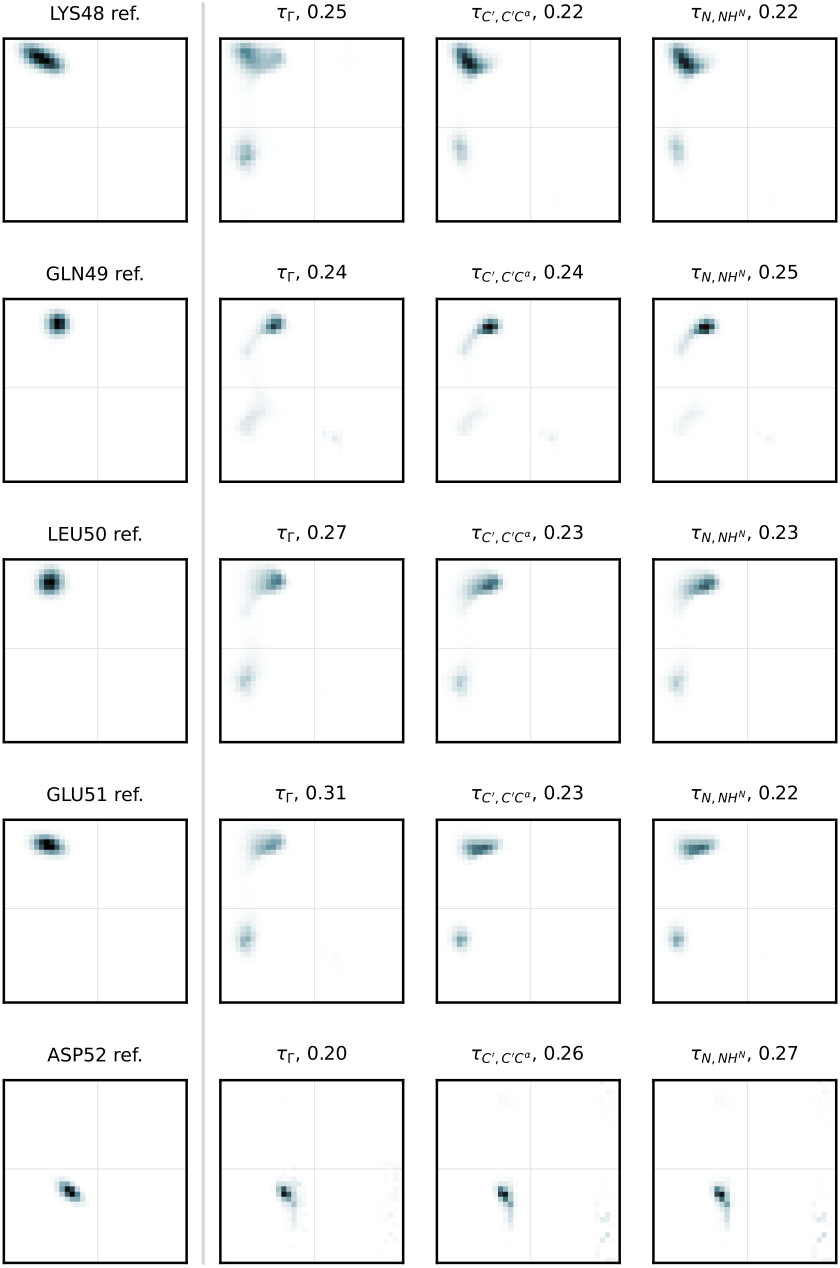

Fig. 6: UBQ ϕ,ψ-distributions, full caption, see below.

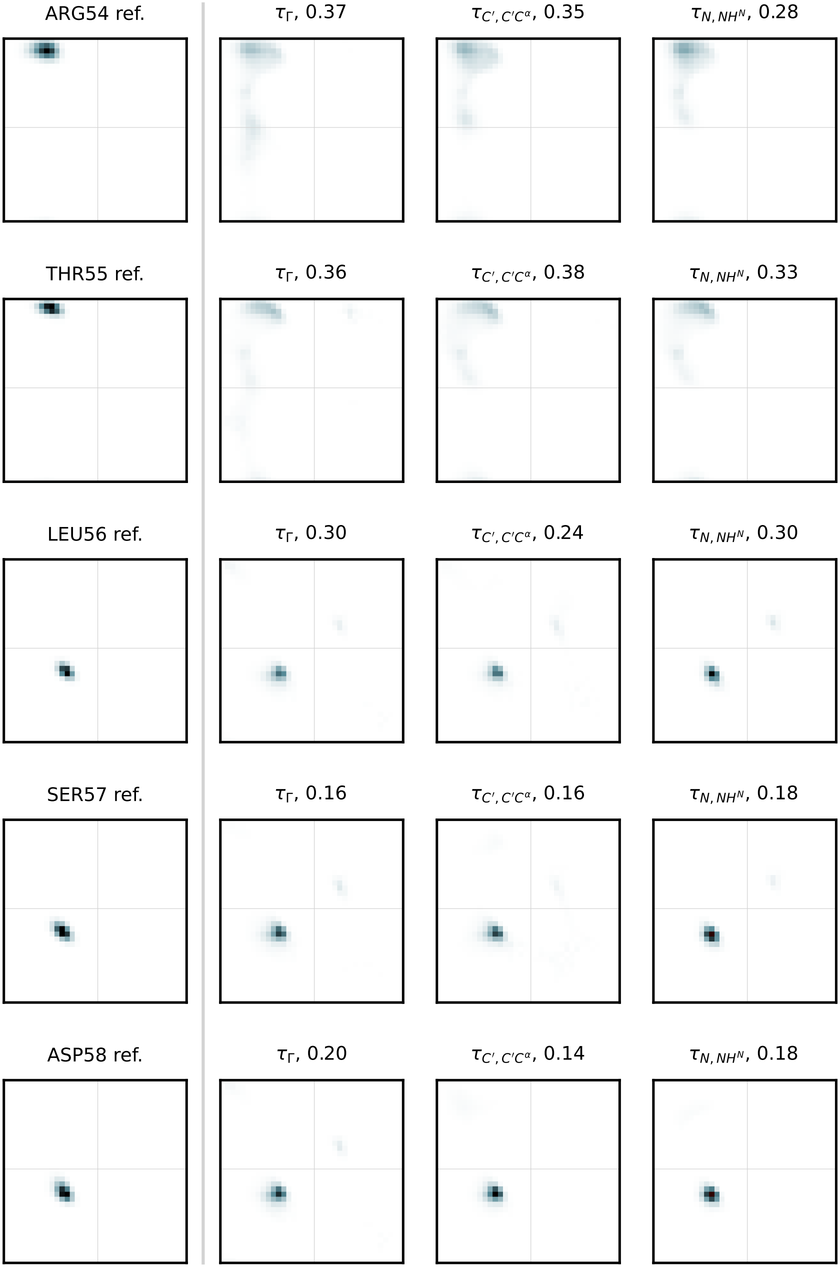

Fig. 6: UBQ ϕ,ψ-distributions, full caption, see below.

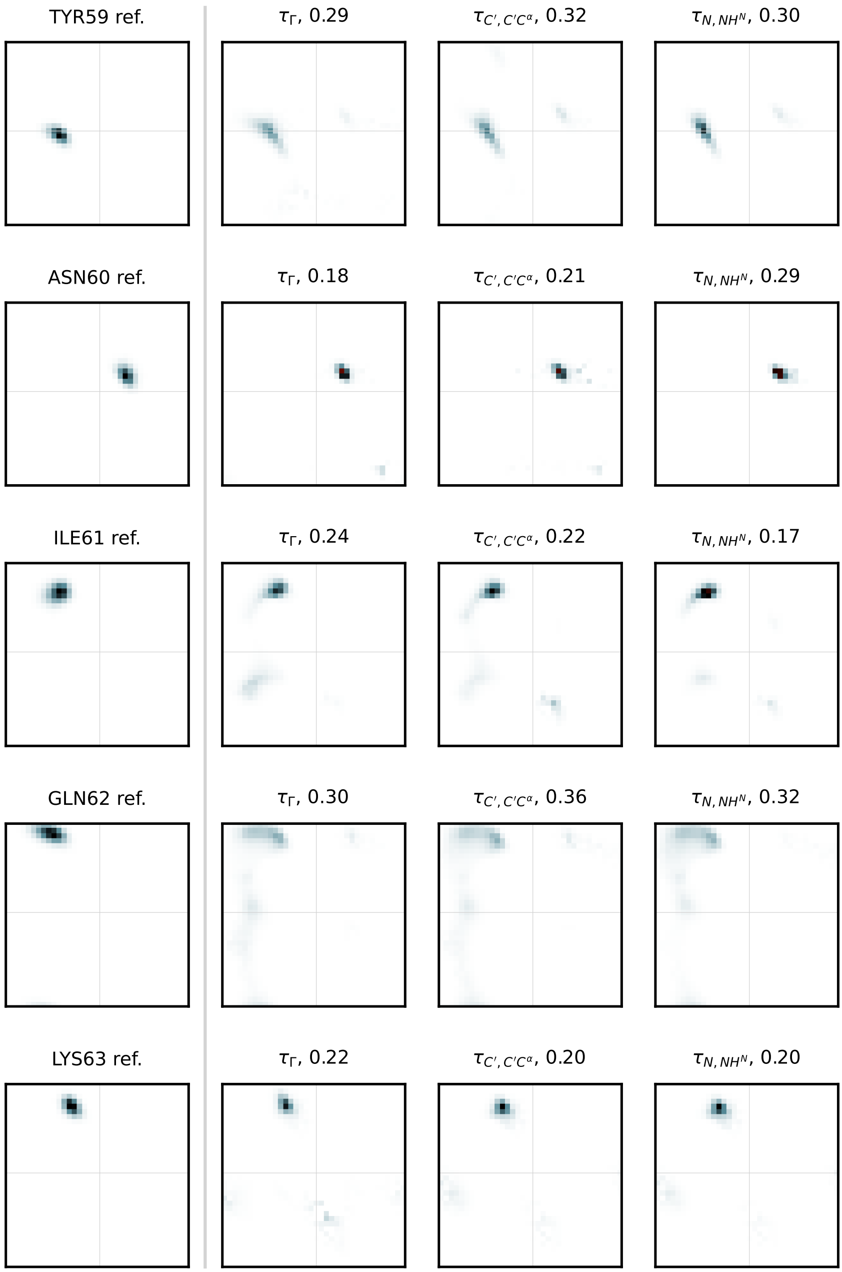

Fig. 6: UBQ ϕ,ψ-distributions, full caption, see below.

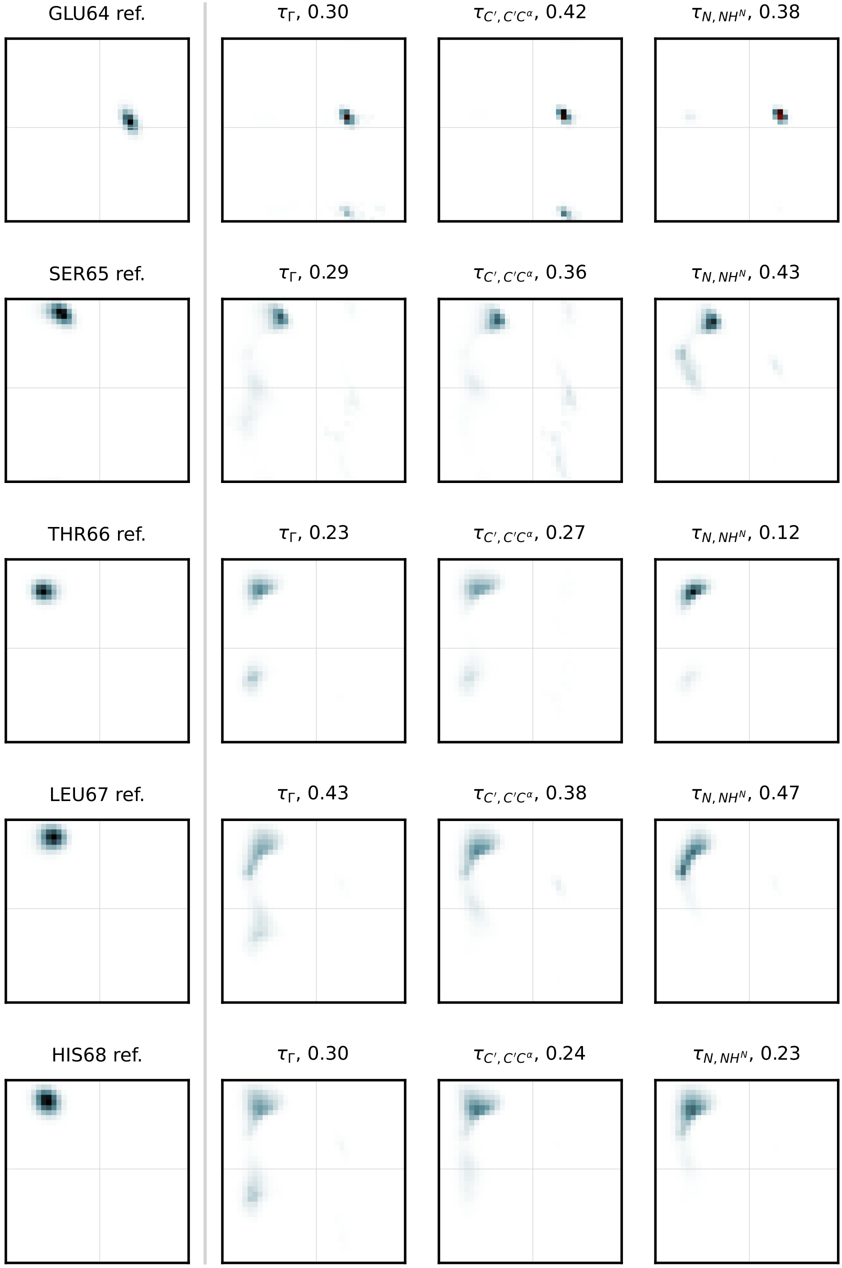

Fig. 6: UBQ ϕ,ψ-distributions, full caption, see below.

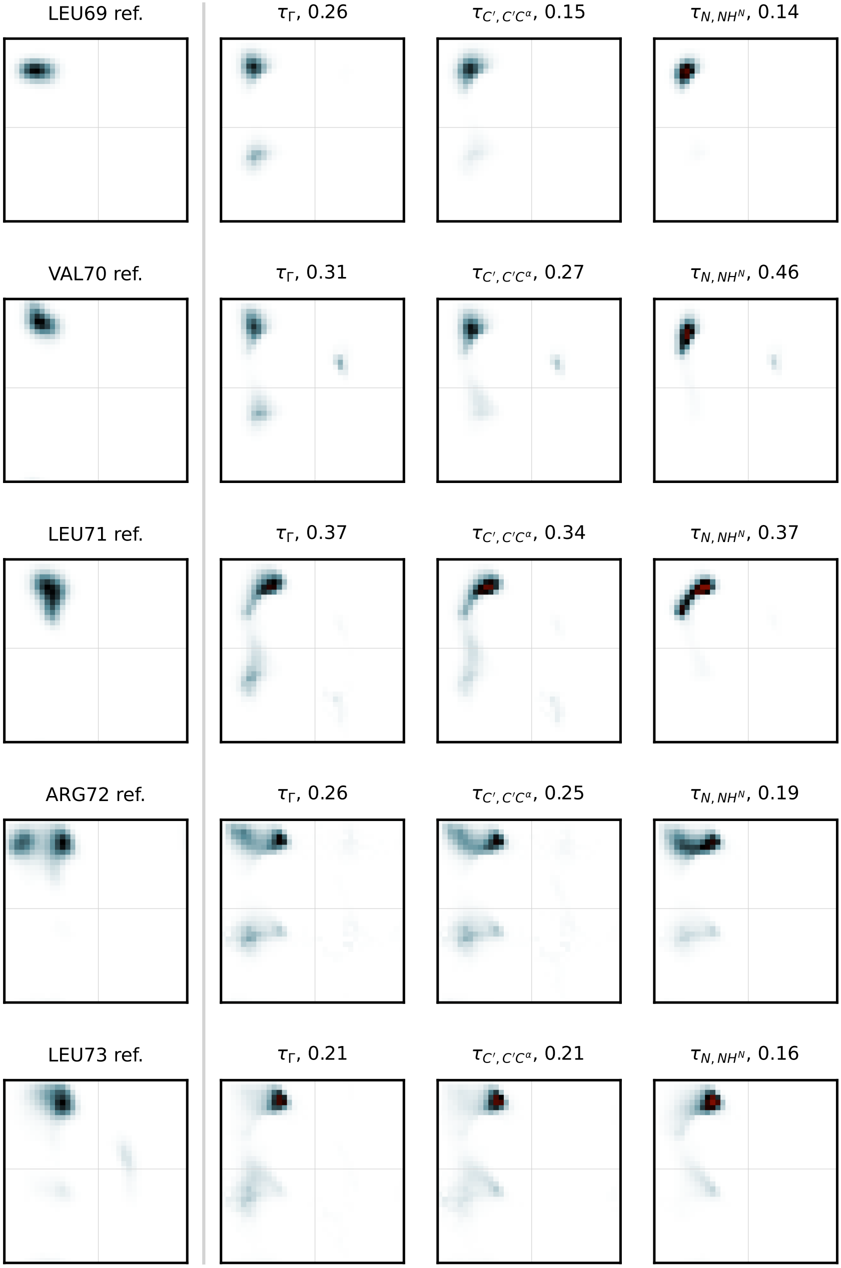

Fig. 6: UBQ ϕ,ψ-distributions, full caption, see below.

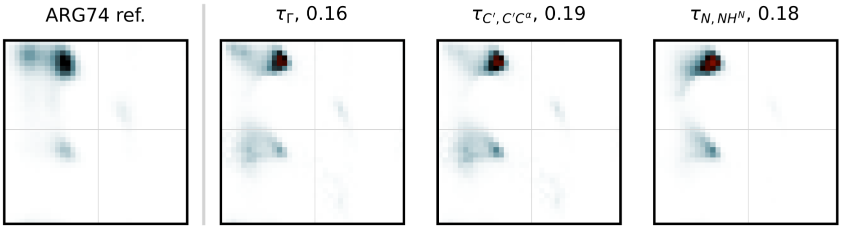

Fig. 6: UBQ ϕ,ψ-distributions. Each line represents a residue. From left to right: reference distribution $\boldsymbol{r}_{\phi,\psi}$ extracted directly from simulation; distribution predicted from rates together with the rates' own correlation time τ_Γ; with $\tau_{C',C'C^\alpha}$; with τ_{N,NH^N}. For each residue, the range of the color scale is defined by the range of $\boldsymbol{r}_{\phi,\psi}$ (see main paper). For all predictions the Jensen-Shannon divergence with respect to $\boldsymbol{r}_{\phi,\psi}$ is provided (see Methods). The mean JS divergence over all residues is similar for all three dynamical proxies at $\sim$0.26.
